# Interpretable Machine Learning for Evaluating Nanogenerators’ Structural Design

**DOI:** 10.1021/acsnano.5c02525

**Published:** 2025-04-07

**Authors:** Chi Han, Mingyu Jin, Fuying Dong, Pengchong Xu, Xinnian Jiang, Sheling T. Cai, Yuanwen Jiang, Yongfeng Zhang, Yin Fang, Simiao Niu

**Affiliations:** † Department of Biomedical Engineering, 242612 Rutgers University, Piscataway, New Jersey 08854, United States; ‡ Department of Computer Science, Rutgers University, Piscataway, New Jersey 08854, United States; § Department of Electrical and Computer Engineering, Rutgers University, Piscataway, New Jersey 08854, United States; ∥ Department of Electrical and Computer Engineering, 14589 University of Illinois Urbana−Champaign, Urbana, Illinois 61801, United States; ⊥ Department of Materials Science and Engineering, University of Pennsylvania, Philadelphia, Pennsylvania 19104, United States; # School of Chemical and Biomedical Engineering, Nanyang Technological University, Singapore 637459, Singapore

**Keywords:** triboelectric nanogenerator, interpretable machine learning, self-powered systems, structural optimization, surrogate models

## Abstract

The limited battery life in modern mobile, wearable, and implantable electronics critically constrains their operational longevity and continuous use. Consequently, as a self-powered technology, triboelectric nanogenerators (TENGs) have emerged as a promising solution to this. Traditional approaches for evaluating TENG structural design typically require manual, repetitive, time-consuming, and high-cost finite element modeling or experiments. To overcome this bottleneck, we developed a fully automated platform that leverages machine learning (ML) techniques. Our framework contains an artificial neuron network-based surrogate model that can provide accurate and reliable performance predictions for any structural parameters and a TreeSHAP interpretable ML model that can generate precise global and local insights for TENG structural parameters. Our platform shows broad adaptability to multiple TENG structures. In summary, our platform is an integrated platform that utilizes interpretable ML techniques to solve the complex multidimensional TENG structural evaluation problem, marking a significant advancement in TENG design and supporting sustainable energy solutions in mobile electronics.

## Graphical abstract

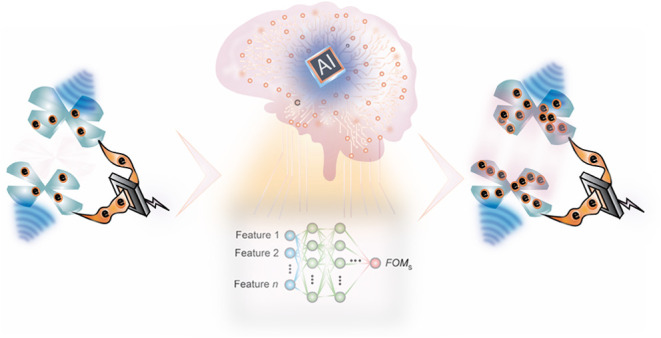

## Introduction

The energy source has become a critical bottleneck for modern mobile, wearable, and implantable electronics, significantly limiting their operational lifetime and continuous usage, particularly in applications like chronic disease monitoring.^1–3^ Besides, the limited battery life of smartphones and smartwatches often disrupts user experience, and replacing batteries in implantable devices, such as pacemakers,^4^ presents significant challenges. As a result, self-powered energy harvesting technologies have emerged as a promising solution to this problem, allowing devices to operate independently without relying on external power sources. Among the various energy harvesting technologies, such as mechanical energy harvesting,^5–9^ solar cells,^10^ thermoelectric cells,^11^ and electromagnetic energy harvesting,^12,13^ triboelectric nanogenerators (TENGs), which leverage surface phenomena at the micro- and nanoscale, have gained significant attention due to their advantages, including high power,^14,15^ high efficiency,^16–18^ broad material selection that supports nanoengineering,^19–22^ and high adaptability.^23–26^ Currently, the maximum power density of TENGs can reach up to 10 MW/m^2^.^27^ Therefore, TENGs have shown their capabilities in broad applications, including ocean wave (blue energy) harvesting,^28,29^ biomechanical energy harvesting,^30–32^ ultrasound energy harvesting to drive implantables,^33–35^ vibrational energy harvesting,^36–38^ wind energy harvesting,^39^ etc. Scientists have continuously pursued the structural and material optimization to further enhance the device performance for broader applications.^40–43^ Specifically, regarding TENG output improvement, researchers have pioneered in bringing up the capacitance model for various TENG fundamental modes,^44,45^ introducing the TENG figure of merits^46^ and studying the load effects of TENGs.^44,47^ These studies have significantly deepened our understanding of the underlying mechanisms for these devices.

However, despite some promising results and models having been developed, previous studies^48–50^ encountered significant challenges in improving TENGs’ output, since TENGs are always a complex electrostatic system containing multiple structural parameters, and most of the TENG systems lack analytical solutions. Traditional methods^44,45,50–52^ for TENG structural evaluation typically require finite element modeling (FEM) or experiments with numerous structural parameters at high sampling densities. Besides, these methods often involve manual, complicated, repetitive, and time-consuming adjustments on those parameters, leading to substantial experimental and computational costs. Moreover, evaluating the impact of various parameters on TENG performance has largely depended on the subjective judgment of researchers, potentially overlooking globally optimal solutions. Therefore, given that the TENG structural evaluation is a high-dimensional, nonlinear, and sophisticated problem, there is a critical need for a generalizable and highly efficient evaluation mechanism to guide the structural parameter design of TENGs.

To fulfill the aforementioned technological gap, in this work, we have developed an automated, comprehensive, and efficient machine learning (ML)-based framework that is able to provide precise global and local insights to perform TENG structural optimization (Figure 1a). Our framework is comprised of three components, an artificial neuron network (ANN) based prediction model, a XGBoost-based global surrogate prediction model, and a treeSHAP-based explainer (Figure 1b). First, the ANN-based prediction model is designed to accurately predict the TENG’s performance for untested structural parameters with minimal computational cost, thereby significantly enhancing the efficiency and effectiveness of TENG performance evaluations. However, as a black-box model, ANN model’s inputs, namely structural parameters are challenging to be interpreted. Given this challenge, it becomes essential to adopt a post hoc interpretation strategy that can elucidate the contributions of each structural parameter, among which SHAP was selected, as it delivers reliable and additive feature attributions that offer both precise local insights and robust global interpretability. However, directly applying SHAP to the ANN would be computationally intensive and insufficient for capturing the complex interactions among features. Therefore, to overcome this, a treeSHAP-based explainer was developed to quantitatively evaluate the independent and interactive importance of input structural parameters. To support the treeSHAP analysis, we construct a XGBoost-based precise surrogate prediction model from extensive ANN outputs and use treeSHAP to explain the XGBoost surrogate model. This explanation model assessed structural parameters from both global and local perspectives, derived new combinations of structural parameters based on feature interactions and provided insights on the structural optimization approach. Compared to other ML approaches used in engineering, such as models based on Bayesian Optimization,^53^ ML-assisted inverse design,^54^ or black-box optimization methods,^55^ our pioneering integration of surrogate models and interpretable ML will not only streamline the optimization process but also offers clear and actionable insights into the individual significance and interactions of various structural parameters, allowing researchers to understand the influence of each parameters, identify potential new feature combinations, discover novel physics mechanisms, and achieve optimal performance more efficiently.

**Figure 1:**
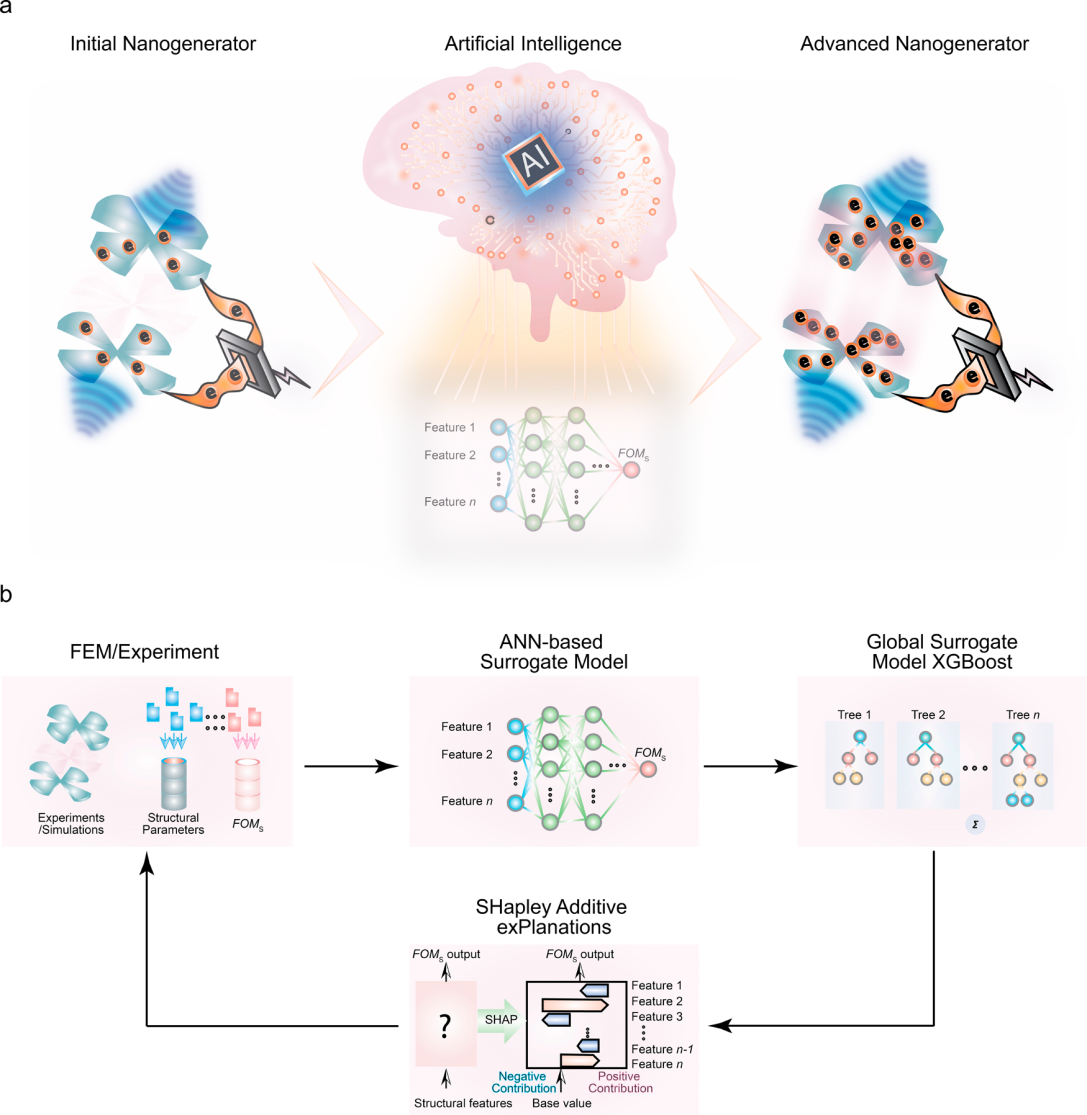
(a) Illustration and (b) flowchart of our TENG structural design evaluation system’s working process, in which surrogate models and interpretable machine learning are involved.

## Results/Discussion

To illustrate the effectiveness of our novel interpretable ML-based approach, we test it on two commonly used TENG models. The first model examined here is the disk TENG model. Disk TENG model is a widely used configuration that can generate high potential outputs.^56^ However, disk TENG is a complicated 3-dimensional model lacking analytical solutions. Therefore, its structural optimization strategy remains a challenging problem and is an ideal candidate to showcase the capability of our interpretable ML-based approach.

The first step to perform interpretable ML to analyze the TENG’s performance is to build the data set, in which the input variables are the structural parameters *d, h, n*, and *ε* (Figure 2a), and the target output variables are the structural figure of merit (*FOM*_S_).^46^ For the input and output variables selection criteria, on the input side, considering that the cross-sectional area is linearly related to the output of the TENG,^46^ the radius of the disk is not included as a structural parameter. Since the scaling of TENG size can only lead to proportional scaling of performance, we choose to use scaling coefficients in TENG models during the actual optimization work to determine the best output performance of the TENG under a given size. On the output side, *FOM*_S_ was selected since it is the gold standard to evaluate the TENG performance and independent with the load behavior.^46^ We have used COMSOL to perform 3D FEM simulations (Methods, Supporting Information Note 1 and Figure S1) to determine the short-circuit transferred charge and capacitance,^57^ then calculation of *FOM*_S_ (eq S2). By sweeping the structural parameters, we obtained a ML data set containing 1260 data points. The use of FEM instead of experiments is justified, as our previous studies have proved that the simulation results align closely with experimental data.^45,57^

**Figure 2:**
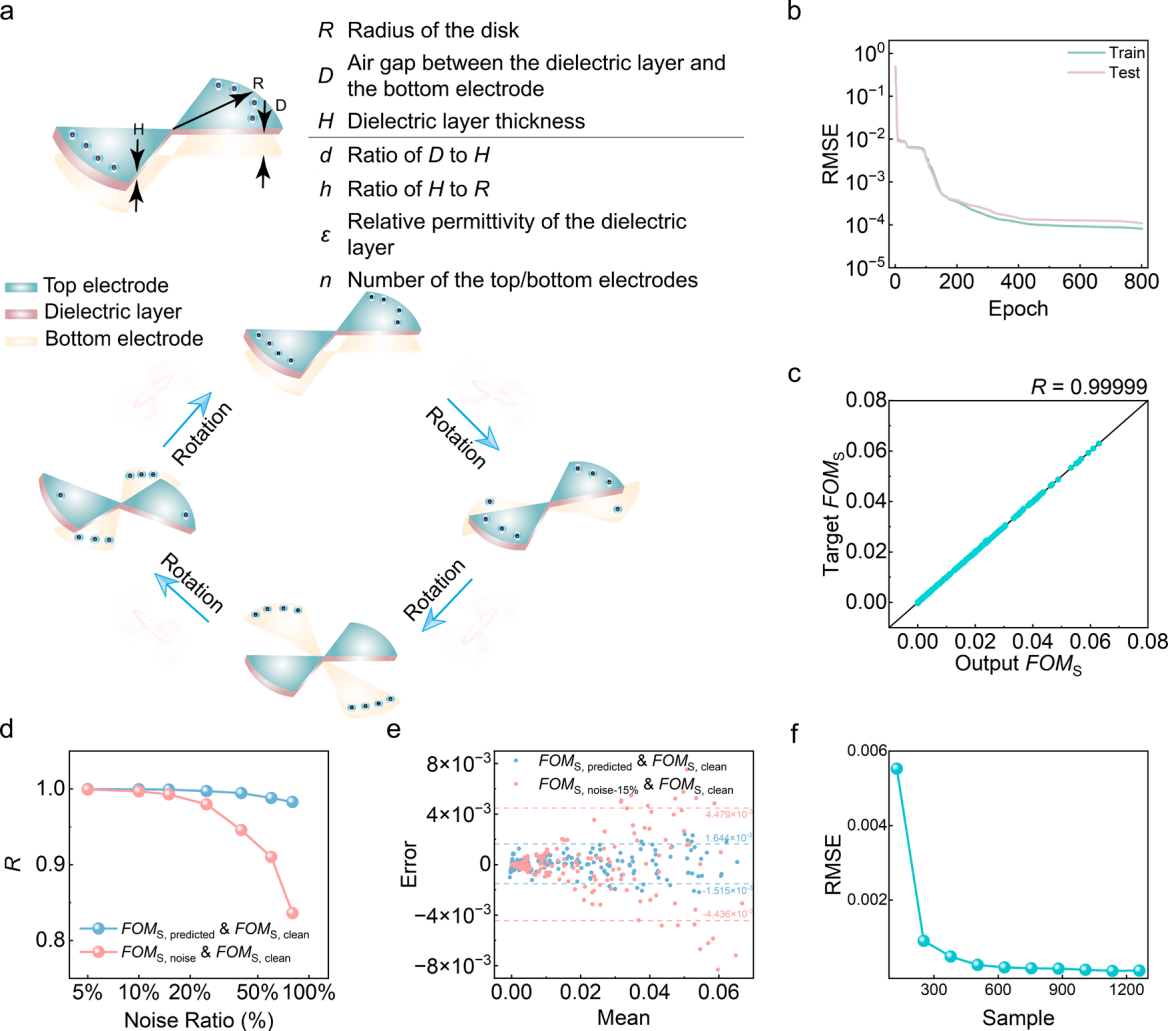
(a) Demonstration of disk TENG working mechanism and its structural parameters; (b) relationship between root-mean-square error (RMSE) loss and epoch plot of the train and test data sets on disk TENG, and (c) regression plot (with Pearson correlation coefficient *R*) of the test data set on disk TENG; (d) Pearson correlation coefficient *R* between the predicted values and the original values without added noise and between the original values and the added noise values, obtained by training on the data set with added noise, with noise ratio ranging from 0% to 80%; (e) Bland-Altman plot between the predicted values and the original values without added noise, and between the original values and the 15% added noise values, obtained by training on the data set with 15% added noise; (f) RMSE loss value of ANN-based surrogate model trained on the reduced data set.

### ANN-Based Surrogate Model for *FOM*_S_ Prediction

Once the data set was obtained, we have developed an ANN-based surrogate model (Figure 1b) to perform prediction of the TENG performance. With the ANN, we obtained significant time savings compared to repeating FEM simulations or performing physical experiments in complicated 4D design space. The ANN model was chosen because it offers much higher accuracy (lower RMSE loss and higher correlation coefficient) compared to other commonly used prediction models such as random forest, XGBoost, and support vector regression (Table S1). A shallow ANN model with 2 hidden layers was constructed, and each layer contains 9 neurons to reduce model complexity and prevent overfitting. To further provide improved generalization on unseen data and prevent potential overfitting, Bayesian regularization backpropagation is applied for the ANN here.^58^ Our ANN model demonstrates outstanding performance, characterized by high accuracy without overfitting, noise robustness, and high data efficiency. First, during the training process, the ANN-based surrogate model demonstrates rapid convergence of the RMSE loss, achieving exceptionally low convergence values without any overfitting observed (Figure 2b). Our prediction also exhibits a strong correlation with the original test data set (Figure 2c), indicating extremely high prediction accuracy (*R* = 0.99999). Second, the ANN-based surrogate model maintains a robust noise performance. To prove the noise robustness, we purposely added random white noise into the training data set and compare the error between model output and the clean data, with the error between the contaminated data and the clean data. When the noise intensity ranges from 5% to 80%, even trained with the contaminated data set, compared to the contaminated data, our ANN prediction achieves stronger correlations with the clean data (Figure 2d and Figure S2). This demonstrates that our ANN model is capable of grasping the primary trend inside the data set and automatically suppressing the random white noise. Specifically, Figure 2e reveals that with a 15% noise level added to the original data set, the 95% confidence interval between the predicted and clean values is approximately one-third of the 95% confidence interval between the contaminated values and clean values. This noise robustness is important and useful since FEM and experimental data always contain noise, and using this ANN helps remove the noise influence. Third, our ANN model demonstrates a remarkable data efficiency. Even when significantly reducing the data set amount from 1260 (original data set) to 252 (20% percent original data set), the ANN model maintains high predictive accuracy. This accuracy is highlighted by the model’s ability of producing RMSE and Pearson correlation coefficients that are nearly identical to those obtained with the full data set (Figure 2f and Figure S3). This ability to maintain performance with limited data is crucial for applications (FEM or experiments), where data collection is expensive or time-consuming. These findings highlight the ANN-based surrogate model’s accuracy, noise robustness, and data efficiency, making it a valuable tool for reliable predictions even in the presence of noise and other uncertainties in the data set.

Using the ANN-based surrogate model allows for global output prediction in the 4D complex design space, providing a more intuitive understanding of how different structural variables influence the *FOM*_S_. With a limited and discrete set of FEM results (Figure 3a), the ANN-based surrogate model can generate continuous predictions of *FOM*_S_ (Figures 3b,c and S4–S6), making the impact of each structural features on *FOM*_S_ more comprehensible and evident. Specifically, Figure 3b illustrates the influence of the other three structural variables on the output *FOM*_S_ when *d* = 0. The scatter plots with contours reveal that variables *n* and *h* exhibit an explicit nonmonotonic relationship with other features (Figures S4–S5), while variables *ε* and *d* demonstrate a distinct monotonic relationship with the other variables (Figures S4–S5 and S6d). During the process of comprehensively examining the contour plots of *FOM*_S_ predictions for all structural parameters, it is observable that the relationship between *n* and *h* is more complex than other variables. From Figure 3c, it is observed that at *d* = 0, the contour line in that plot is close to straight lines parallel to the diagonal. This shows that the product of *nh* plays a primary role in determining the *FOM*_S_. Additionally, as *nh* increases, *FOM*_S_ initially rise and then fall, indicating a nonmonotonic relationship between *nh* and *FOM*_S_, namely an optimum *nh* when *FOM*_S_ reaches the highest value (Figures S6a–c). The observed dependence of the structural parameters on *FOM*_S_ can be explained well by the underlying physics. Known that the parameter *n*, which represents the total number of electrodes, is inversely proportional to the arc length of the substructure, the product *nh* therefore is directly proportional to the ratio of the dielectric thickness to the arc length of the substructure, representing the aspect ratio of the substructure cross-section in disk TENGs. This aspect ratio significantly influences the edge effect within disk TENGs.^57,59^ When the aspect ratio is sufficiently large and the air gap is small, each individual disk unit approximates the ideal infinite-size parallel capacitor model, resulting in near 100% charge transfer efficiency once full lateral separation is reached. However, as this aspect ratio decreases, the infinite-size parallel capacitor model no longer applies, leading to a significant drop in charge transfer efficiency due to the edge effect in sliding/grating TENGs. This edge effect is the primary reason for the observed nonmonotonic behavior. For the individual parameters, let us consider *n* and *h* first. The *FOM*_S_ has a term of *n* in the numerator (eq S2), which explains why *FOM*_S_ increases initially as *n* increases. However, once *n* becomes sufficiently large, the substructure aspect ratio decreases, the edge effect becomes dominant, and the charge transfer efficiency drops, causing *FOM*_S_ to decrease. For parameter *h*, when *h* increases initially, the infinite-size parallel capacitor model still holds, so the charge transfer efficiency remains approximately 100%. Since the open-circuit voltage is linearly proportional to *h*,^57^ the *FOM*_S_ will increase. However, as *h* continues to increase, the edge effect becomes dominant, leading to a decrease of the charge transfer efficiency and *FOM*_S_. Therefore, the combination of *nh* is the most important structural parameter to determine *FOM*_S_. Besides *n* and *h*, *d* and *ε* will lead to a monotonic decrease of *FOM*_S_. The increase of *d* will lead to significant amount of the charge left at the bottom electrode at the initial overlapping state, considering its initial contact separation process.^45^ Therefore, it reduces the total amount of transferred charges during rotation. For the variable *ε*, an increase in *ε* will lead to an increase in capacitance, which, in turn, will result in a decrease in *FOM*_S_. This analysis provides crucial insights into the impact of each variable and variable interactions on *FOM*_s_, aiding in the optimization of system parameters to enhance overall performance.

**Figure 3:**
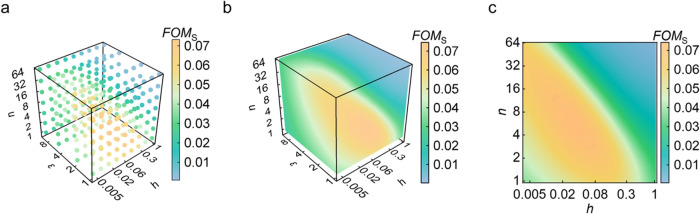
(a) 3D plot of *FOM*_S_ of the original data set when *d* = 0 and (b) the data set generated by ANN-based surrogate model on the *h*-*ε*-*n* coordinate when *d* = 0; (c) 2D plot of *FOM*_S_ of the data set generated by the ANN-based surrogate model on the *h*-*n* coordinate, when *d* = 0 and *ε* = 1.

### TreeSHAP for Quantitative Structural Parameters’ Evaluation

Above we showed that an ANN model has been developed to predict the TENG output and determine the influence of each structural parameter through observation of the *FOM*_s_ plot. However, this is a qualitatively method, and the ANN itself is a black-box model. To overcome this drawback, we have integrated an interpretable ML model with this ANN so that the impact of each input parameter on *FOM*_S_ can be quantitatively reflected (Figure 1b). The interpretable ML model requires a to-be-explained model, which can be either the ANN model we developed previously or a global surrogate model that predicts *FOM*_S_. Among the interpretable ML models, Shapley Additive Explanations (SHAP) derives from the Shapley value in cooperative game theory.^60^ Its primary goal is to fairly distribute the contribution of each feature to a model’s prediction. SHAP completes the evaluation by measuring how a model’s prediction changes when adding or removing each feature under different combinations, thus ensuring an additive and consistent allocation of contribution across all features. For each instance, SHAP calculates a separate SHAP value for each feature. By visualizing these values (e.g., force plot), we can see which features have the greatest impact on the prediction in that specific context. Across multiple instances, aggregating SHAP values reveals how strongly each feature influences the model’s predictions on average, highlighting both global and local feature importance. For each SHAP value, a positive SHAP value means a given feature is pushing the model’s prediction higher for that instance, whereas a negative SHAP value indicates the feature is pulling the prediction lower. This sign-based interpretation shows whether each feature reinforces or diminishes the prediction in individual cases. By averaging or summarizing SHAP values over a data set, we can derive a measure of overall feature importance, reflecting how much each feature contributes to the model’s predictions in the aggregate. This approach provides a straightforward way to compare features against one another in terms of predictive strength, as well as a transparent additive breakdown that clarifies each feature’s role. Here, the SHapley Additive exPlanations (SHAP), specifically, treeSHAP was utilized as the explainer, since it provides explanations that closely resemble those obtained by directly interpreting the ANN, while significantly enhancing computational efficiency.^61^ Additionally, it allows for a more intuitive observation of the interactions between input features.^61^ Consequently, to accompany the treeSHAP-based explainer, a tree-based XGBoost global surrogate model was selected to facilitate this interpretation. Specifically, the XGBoost global surrogate model was obtained by feeding 810,000 sets of the *FOM*_S_ data set generated from the ANN. This approach enhances the ability of the constructed XGBoost global surrogate model to more accurately replicate the behavior of the ANN. Grid search was applied here to fine-tune the hyperparameters of the XGBoost regressor, enabling its outstanding performance (Figures S7–S8). After hyperparameter tuning, the number of gradient boosted trees was set to 300, the maximum tree depth for base learners was 14, and the boosting learning rate was 0.38125.

The treeSHAP not only evaluates the importance of individual features but also provides insights into how pairs of features interact to influence the model’s output. Through the interpretation of the XGBoost model using treeSHAP, we observed that, based on Mean Absolute SHAP values, variable *d* emerged as the most dominant feature (Figure 4a). This corresponds well with the previously mentioned physics explanation. Further investigation into the potential interactions between variables *n* and *h* on the ANN-based surrogate model led us to analyze the global surrogate model’s interaction heatmap (Figure 4b). Despite *n* and *h* not being the most influential individual features, their interaction was significantly more impactful compared to that of other feature interactions (Figure 4c). This phenomenon highlighted the importance of the interaction between *n* and *h*. Consequently, based on the findings in Figure 3c, we introduced the variables *nh* and *n/h* into the data set. After retraining and interpreting the global surrogate model after first interaction, it became evident that variable *nh* surpassed variable *d*, becoming the most dominant feature (Figure 4d), consistent with our hypothesis in our previous discussion regarding the parameter *nh*’s physical understandings.

**Figure 4:**
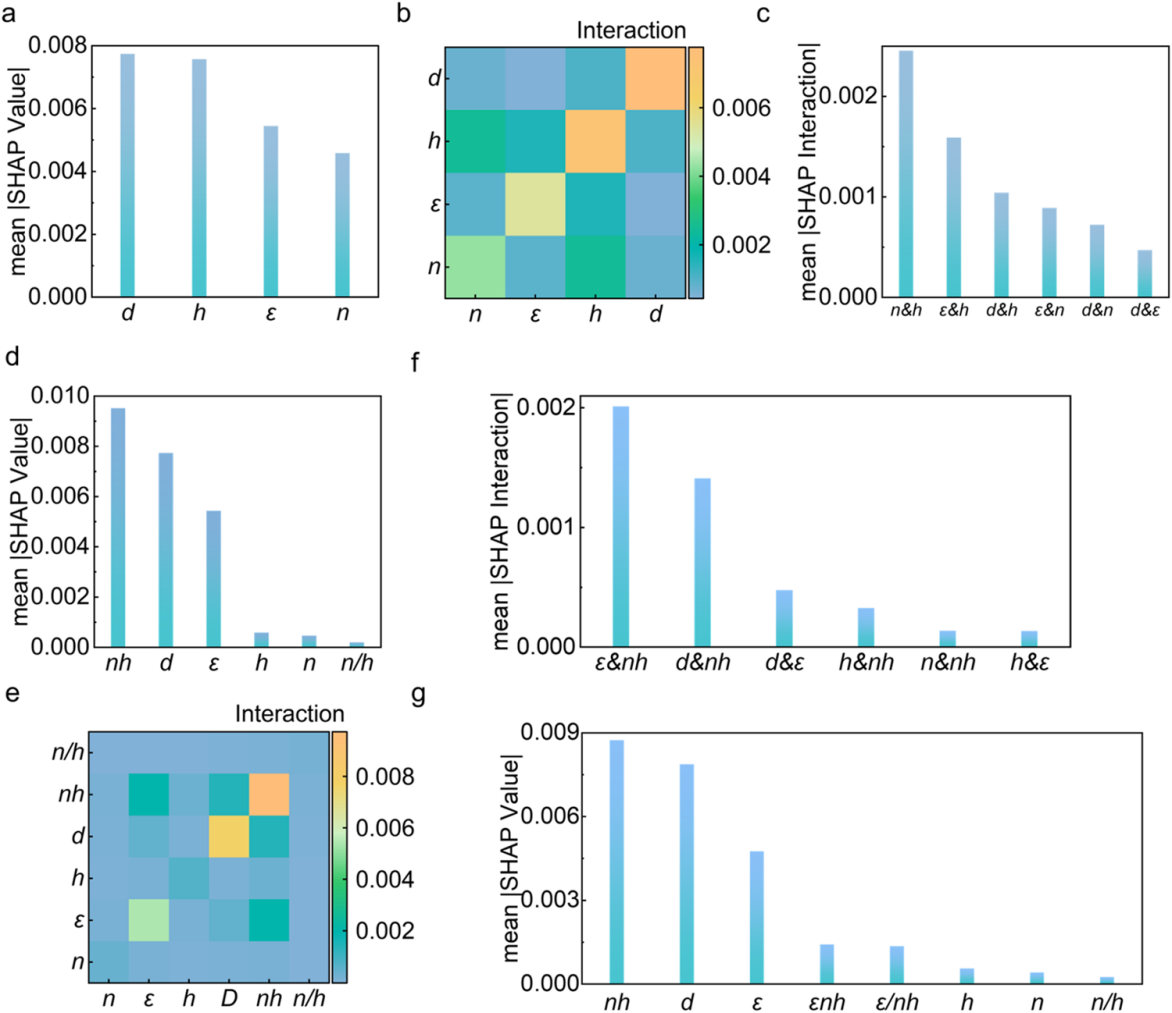
(a) Mean absolute SHAP values plot and (b) the mean absolute SHAP value interaction heatmap of original features on the original data set; (c) mean absolute SHAP values plot of interaction features on the original data set; (d) mean absolute SHAP values plot and (e) the mean absolute SHAP value interaction heatmap of new features on the data set after first interaction; (f) mean absolute SHAP values plot of new interaction features on the data set after first interaction; (g) mean absolute SHAP values plot of new features on the data set after second interaction.

Similarly, we examined the interaction heatmap after the first iteration (Figure 4e) and found that *ε* and *nh* were the most significant interaction features (Figure 4f). However, when incorporating this interaction as an independent feature into the data set for a second iteration, its contribution to the prediction of *FOM*_S_ was negligible (Figure 4g). The output from the ANN-based surrogate model also indicated that there was no strong interaction between variables ε and *d* with *nh* (Figure S6a-c). The findings underscore the critical role of the *nh* feature in the model, reinforcing its significance in subsequent studies and analyses.

Focusing on mean absolute SHAP values provides a valuable global perspective on feature importance but can obscure the details of feature contributions. This approach makes it difficult to capture how feature polarity influences *FOM*_S_, and overlooks local variations in feature impact. Therefore, analyzing the distribution of SHAP values and examining local feature contributions are crucial to fully understand the nuances of the model’s behavior between features.

The SHAP summary plot of the original data set provides an excellent perspective for analyzing the positive and negative contributions of feature magnitudes to *FOM*_S_ (Figure 5a). It clearly shows a monotonic relationship between the magnitudes of features and *d* and their SHAP values, in which smaller values of *ε* and *d* contribute more positively to *FOM*_S_. In contrast, features *n* and *h* exhibit noticeable nonmonotonic behavior. These trends are consistent with the prediction of the ANN-based surrogate model. To further analyze the impact of each parameter, we turned to a local analysis by selecting groups with high and low *FOM*_S_ values (Table S2). In this local analysis, it becomes evident that variable *d* plays a dominant role in higher *FOM*_S_ values (its SHAP value is the highest among the 4 input parameters), while variable *h* is the primary contributor to lower *FOM*_S_ values (its SHAP value is the smallest among the 4 input parameters) (Figure 5b). The combined insights from the SHAP summary plot and the local analysis provide a comprehensive understanding of how different features influence the *FOM*_S_, allowing for more precise adjustments to optimize the model’s performance.

**Figure 5:**
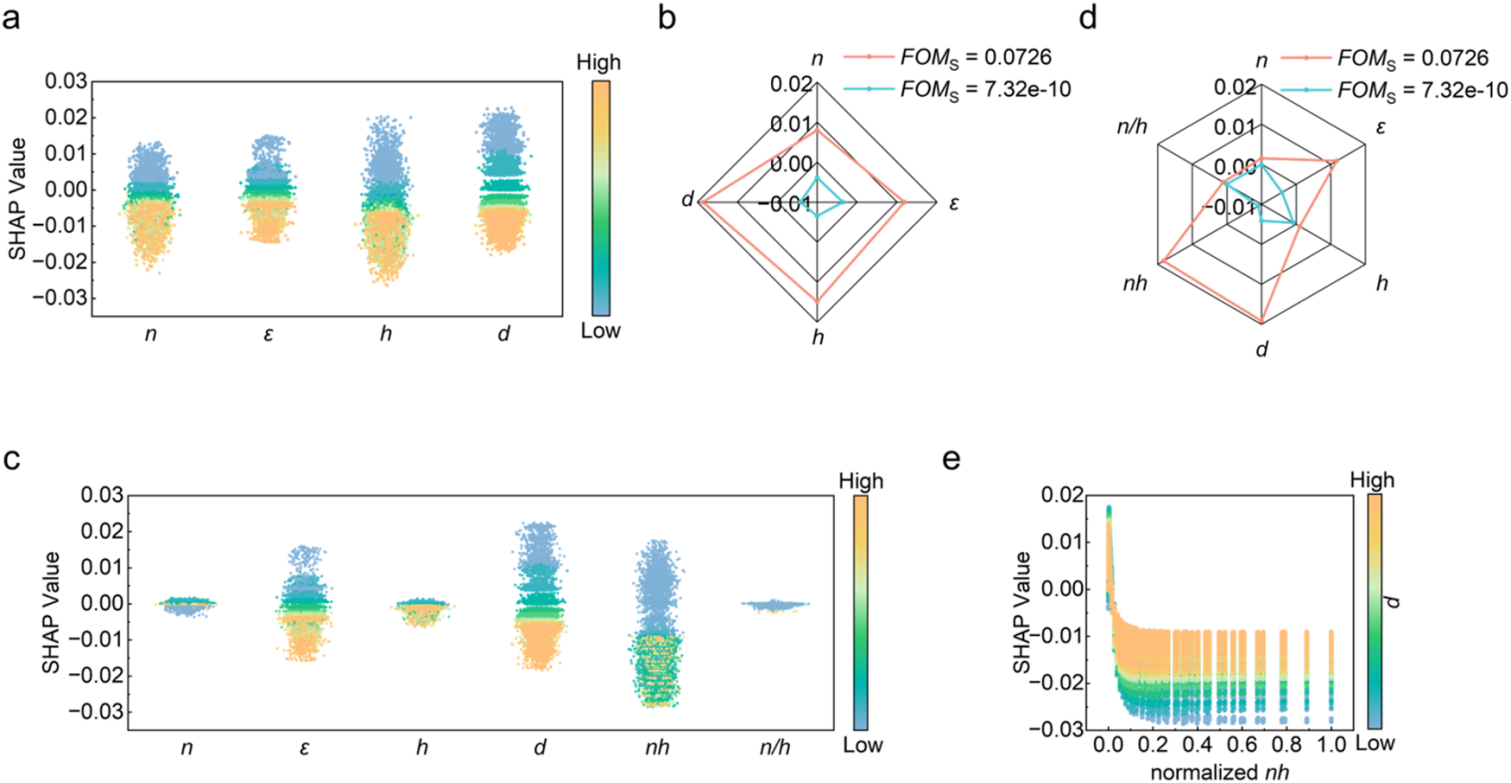
(a) SHAP summary plot and (b) SHAP values of individual interpretation on a high *FOM*_S_ value example and on a low *FOM*_S_ value example for original features on the original data set; (c) SHAP summary plot and (d) SHAP values of individual predictions on a high *FOM*_S_ value example and on a low *FOM*_S_ value example for new features on the data set after first interaction; SHAP dependence plots of (e) the normalized feature *d* interacting with the normalized feature *nh*.

After adding the new features *nh* and *n/h* to the data set following the first interaction, the updated SHAP summary plot reveals that the original variables maintained their previous trends. The newly introduced dominant variable *nh* exhibits more pronounced nonmonotonic behavior with an optimum *nh* contributing the largest *FOM*_S_ (Figure 5c). This observation is also evident in the selected parameters for local analysis (Table S2). In the local analysis, both *nh* and *d* play equally dominant roles in increasing *FOM*_S_ values, while *nh* emerges as the primary contributor to decreasing *FOM*_S_ values (Figure 5d), further confirming that *nh* is the dominant feature.

The previously shown trends of the influence of each structural parameter are further shown by the SHAP dependence plots (Figures S9 and S10). For the *nh* variable, the SHAP dependence plot reveals a distinct maximum value, demonstrating a peak in its influence on *FOM*_S_ (Figures 5e and S10). This peak aligns with the contour plot of *nh* in the ANN-based surrogate model, suggesting a consistent pattern across the different analytical approaches. Furthermore, we observe that as the values of *d* and *ε* increase, the influence of *nh* becomes more stable, indicating that the impact of *nh* weakens (Figures 5e and S10). This stabilization supports the findings from the SHAP summary plot, where the interplay among these variables was initially highlighted.

The consistency between the SHAP dependence plots and the SHAP summary plot strengthens our understanding of the complex relationships between the features and their contributions to *FOM*_S_. It underscores the importance of *nh* as a critical feature, particularly in its interactions with other variables. This comprehensive analysis not only confirms the dominance of *nh* but also provides deeper insights into how changes in one feature can modulate the impact of another, facilitating more informed decisions for model optimization and feature engineering.

Moreover, the consistency in feature importance explanations across different data volumes underscores the robustness of the global surrogate model XGBoost. When using 20% of the original data set, the global surrogate model still identifies the same key features as in the full data set scenario (Figures S11–S12). This indicates that the model’s understanding of feature importance is stable and reliable, even with less data. In scenarios with just 10% of the original data, the model’s capability to pinpoint *nh* as the most important feature after the first interaction further exemplifies its efficiency and reliability. This is critical for iterative model refinement and feature selection processes, where ensuring the accuracy and relevance of identified features can significantly enhance the model’s performance and interpretability.

### Generalizability Demonstration of Our Proposed Explanation Framework

Our developed method that combines ANN and treeSHAP is a general method that can be extended to different categories of TENGs. To further prove the generalizability of this surrogate model, we utilized similar methods to evaluate a spherical TENG model that is designed to harvest ocean wave energy^28^ (Figure S13). We have built the FEM model, assigned proper boundary conditions (Methods and Supporting Information Note S2), and obtained a 7744-data point ML data set by sweeping the 5 structural parameters, including *ε*_shell_, *ε*_ball_, *θ*, *dR*, and *dH* (detailed explanation of each structural parameter shown in Figure 6a and Supporting Information). It should be noted that the ANN model of spherical TENG is retrained from the beginning. The ANN-based surrogate model once again demonstrated exceptionally high predictive accuracy (Figure S14) and data efficiency (Figure S15). Using this model, we generated plots showing the influence of five input features on *FOM*_S_ (Figure 6b–e, Figures S16–S17). It is evident that except for variables *dR* and *dH*, the impacts of the other variables on *FOM*_S_ are monotonic. To quantitatively study the influence of the input parameters on *FOM*_S_, TreeSHAP was also employed in this analysis. Mean absolute SHAP values plot shows that the radius ratio of dielectric ball *dR* is the most dominating feature on the original data set (Figure 6g). The above results are consistent with the physics explanation. First, given the constant tribocharge density at the inner ball surface, the total triboelectric charge is quadratically correlated with the radius of the dielectric sphere. However, as the radius of the dielectric sphere increases, its volume also increases, and the relative distance difference between the center of the inner ball with the two electrodes therefore significantly decreases, leading to a decrease in charge transfer efficiency between the electrodes. At an extreme case, when *dR* reaches the shell’s inner radius, there is no difference between the inner ball’s center with two electrodes, then the transferred charge amount is 0, resulting in *FOM*_S_ being 0 (Supporting Information Note S2). Consequently, the relationship between *dR* and *FOM*_S_ becomes more prominent. From the mean absolute SHAP value interaction heatmap of the spherical TENG’s data set, as the magnitudes of interaction features are much lower than the main features, we stop at the original features (Figure S18).

**Figure 6:**
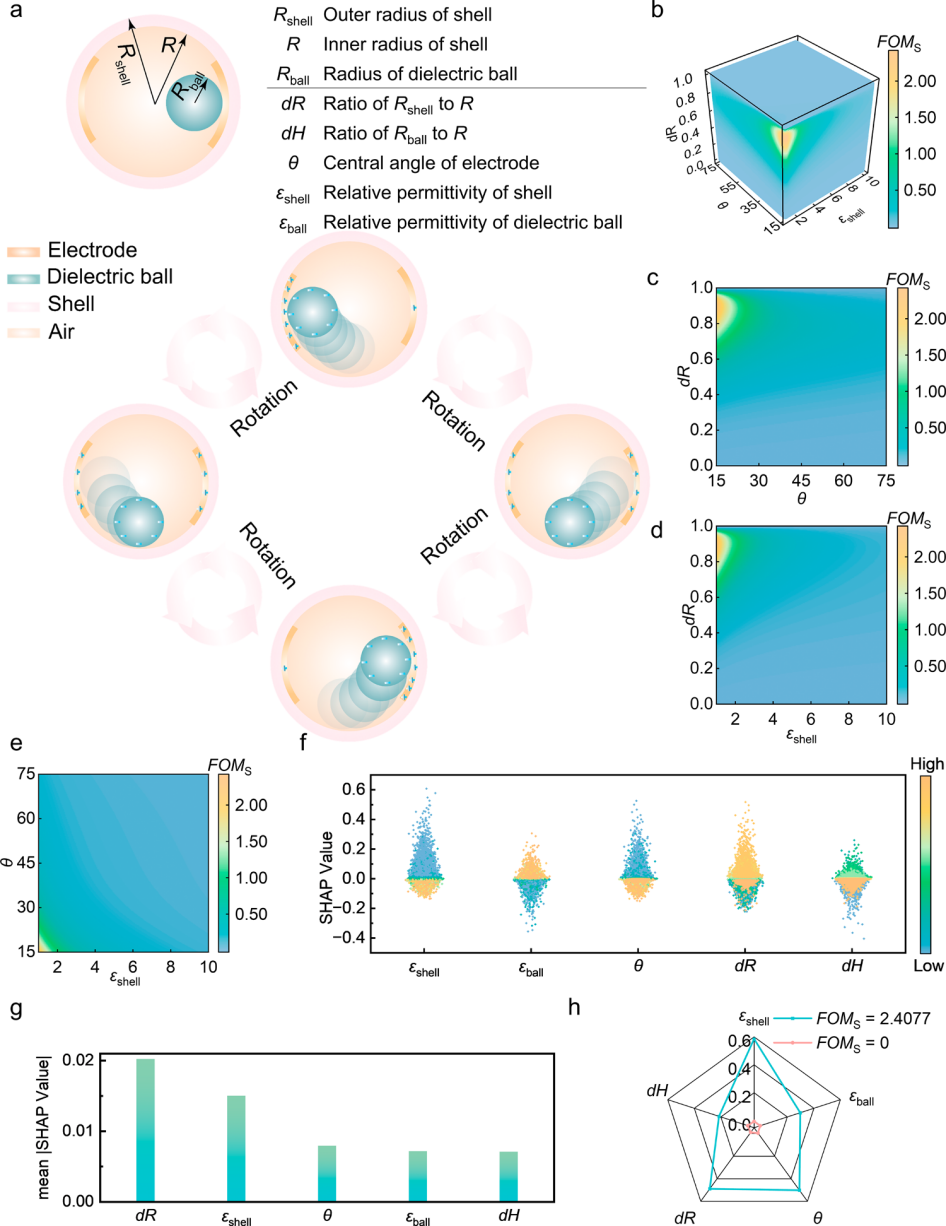
(a) Demonstration of spherical TENG working mechanism and its structural parameters; (b) 3D plot of *FOM*_S_ of the data set generated by ANN-based surrogate model on the *ε*_shell_-*θ*-*dR* coordinate, when *ε*_ball_ = 10 and *dH* = 0.2; 2D plot of *FOM*_S_ of the data set generated by ANN-based surrogate model (c) on the *θ*-*dR* coordinate, when *ε*_ball_ = 10, *dH* = 0.2 and *ε*_shell_ = 1, (d) on the *ε*_shell_-*dR* coordinate, when *ε*_ball_ = 10, *dH* = 0.2 and *θ* = 15, and (e) on the *ε*_shell_-*dR* coordinate, when *ε*_ball_ = 10, *dH* = 0.2 and *θ* = 15; (f) SHAP summary plot, (g) mean absolute SHAP values are plotted and (h) SHAP values of individual interpretation on a high *FOM*_S_ value example and on a low *FOM*_S_ value example for original features on the original data set.

Trends between features and *FOM*_S_ are also observable from the SHAP summary plot (Figure 6f). First, an increase in *ε*_shell_ will lead to a decrease in *FOM*_S_. This is because an increase in *ε*_shell_ leads to an increase in capacitance between the two electrodes since this capacitance is primarily governed by the serial capacitance between each electrode and the grounded outer shell. Second, an increase of *θ* will also lead to a decrease in *FOM*_S_, as this results in a reduction of the effective distance between the two electrodes, ultimately leading to an increase in capacitance and a decrease in the *FOM*_S_. Third, the increase in *ε*_ball_ results in an increase in *FOM*_S_, as this results in an increase in the amount of transferred charges. Fourth, for the nonmonotonicity caused by the feature *dH*, an increase in *dH* leads to a decrease in the capacitance between each electrode and outer grounded shell, which results in an increase in *FOM*_S_. However, since *FOM*_S_ are inversely proportional to the cube of the outer radius of the shell (eq S11), excessive increases in *dH* will result in a decrease in *FOM*_S_. These phenomena can also be observed in the SHAP dependence plots (Figures S19–S20). From a local analysis perspective (Table S3), for achieving a high *FOM*_S_, the selection of *ε*_shell_ needs to be the most cautious, followed by the impacts of *dR* and *θ* (Figure 6h). Moreover, when using 20% of the original data set, the global surrogate model still identifies the same key features as in the full data set scenario (Figure S21). This indicates that the model’s understanding of feature importance is stable and reliable, even with limited amount of data.

## Conclusions

In this work, a comprehensive framework combining a surrogate model and interpretable ML for the structural parameters’ evaluation of sophisticated TENGs is presented. The ANN-based surrogate model for predicting TENG outputs, trained using data sets generated from FEM sparse simulations, demonstrates high accuracy, noise robustness, and data efficiency. By inputting the results from the ANN model, the treeSHAP interpreter addressed the structural parameter importance before and after interaction, providing insights from both global and local perspectives. For disk TENGs, our platform successfully identifies the air gap thickness as the most influential structural parameter before interaction and the individual disk aspect ratio as the most influential parameter after examining the first interaction, which aligns well with the underlying physical mechanisms. To showcase the generalizability of our proposed system, a spherical TENG model was also examined. In this model, the dielectric ball relative radius was identified as the most influential parameter, and no significant interaction between features was observed. By leveraging the ANN-based surrogate model and the treeSHAP interpreter, this study addresses a bottleneck in the structural optimization of TENGs, driving significant advancements in their practical deployment and functionality in real-world applications.

## Methods

### FEM for Disk TENG

The disk TENG model selected here is a conductor-to-dielectric type of TENG, in which the bottom metal acts as not only a triboelectric layer but also the bottom electrode. The surface charge density of the tribo-charges on the dielectric layer is defined as -σ (−1 × 10^–5^ C/m^2^). The total charge of the electrodes can be obtained by multiplying σ by the surface area (*S*) of the tribolayer. As is typical under experimental conditions, the entire structure is surrounded by air. The potential at infinity is chosen as the reference point for the electric potential, set to 0.

The COMSOL simulation for disk TENG here has two stages: start stage, namely, when upper and lower electrodes are exactly overlapping with each other, and end stage, namely, when upper and lower electrodes are completely staggered. For each stage, the parameters utilized are shown in Table S4. For the boundary conditions of the disk TENGs’ simulations here, we assigned the bottom electrode to have the total charge minus the transferred charge (*σS*–*Q*_transfer_) and the top electrode to have a transfer charge amount (*Q*_transfer_). Then, we swept *Q*_transfer_ from 0 to *σS* and use FEM results to calculate the voltage difference between the top and bottom electrodes under different *Q*_transfer_. A linear Voltage-*Q*_transfer_ curve can be obtained, and linear interpolation is used to obtain open-circuit voltage *V*_OC_, *Q*_SC_ and *C* at the start and end stage.^45,57,62^ Then based on these values, we calculated the *V*_OC_ and *Q*_SC_ values under MACRS^62^ and the *FOM*_S_ values can be obtained.

### FEM for Spherical TENG

The spherical TENG model selected here is a conductor-to-dielectric type of freestanding TENG, in which each metal acts as not only a triboelectric layer but also the electrode. The surface charge density of the tribo-charges on the dielectric ball is defined as -σ (−7 × 10^–6^ C/m^2^). The total charge of the system can be obtained by multiplying this value by the surface area of the dielectric ball (4π*R*_ball_^2^, *R*_ball_ represents the radius of the dielectric ball). The space between the inner surface of the spherical shell and the outer surface of the dielectric sphere is filled with air. To accurately simulate the effect of seawater on the spherical TENG, its outer shell was grounded due to the fact that the seawater is electrically conductive.

For each stage, the parameters utilized are shown in Table S5. The COMSOL simulation for spherical TENG here has only one stage, as their end and start stage are symmetrical. Additionally, we utilize a symmetric charge reference state.^62^ In the symmetric charge reference state, one electrode has a charge equal to half of the total charge minus the transferred charge (*Q*_total_/2 – *Q*_transfer_), while the other electrode has a charge equal to half of the total charge plus the transferred charge (*Q*_total_/2 + *Q*_transfer_). Using a symmetrical charge reference state enables reduction of the calculation load by a half. Then, we sweep *Q*_transfer_ from 0 to *Q*_total_/2 and use FEM results to calculate the voltage difference between electrodes under different *Q*_transfer_. A linear Voltage-*Q*_transfer_ curve can be obtained, and linear interpolation is used to obtain open-circuit voltage *V*_OC_, *Q*_SC_ and *C* at the symmetric charge reference state.^44^ Then, based on these values, we calculated the *V*_OC_ and *Q*_SC_ values under MACRS^62^ and the *FOM*_S_ values can be obtained. It is worth noting that since the symmetric charge reference state is used here, we need to multiply the resulting *Q*_SC_ by 2 to get the final *Q*_SC_ under MACRS.

## Supplementary Material





## Notes

Supplementary data and analysis: COMSOL simulation result for Disk TENG/Spherical TENG; further ANN surrogate models’ performance trained by Disk TENG/Spherical TENG; 3D and 2D *FOM*_S_ plots by ANN surrogate models for Disk TENG/Spherical TENG; XGBoost models’ performance trained by ANN surrogate models for Disk TENG; SHAP dependence plot and mean absolute SHAP values plot for Disk TENG/Spherical TENG with no interaction and first interaction, and its data efficiency; *FOM*_S_ derivation process for Disk TENG and Spherical TENG, and other analyses (PDF)
IML_TENG_Code (ZIP)

## References

[1] Jiang Y., Trotsyuk A. A., Niu S., Henn D., Chen K., Shih C.-C., Larson M. R., Mermin-Bunnell A. M., Mittal S., Lai J.-C. (2023). Wireless,
closed-loop,
smart bandage with integrated sensors and stimulators for advanced
wound care and accelerated healing. Nat. Biotechnol..

[2] Shi J., Kim S., Li P., Dong F., Yang C., Nam B., Han C., Eig E., Shi L. L., Niu S. (2024). Active
biointegrated living electronics for managing inflammation. Science.

[3] Long Y., Wei H., Li J., Yao G., Yu B., Ni D., Gibson A. L. F., Lan X., Jiang Y., Cai W., Wang X. (2018). Effective Wound Healing Enabled by Discrete Alternative Electric
Fields from Wearable Nanogenerators. ACS Nano.

[4] Bhatia A., Hanna J., Stuart T., Kasper K. A., Clausen D. M., Gutruf P. (2024). Wireless Battery-free
and Fully Implantable Organ Interfaces. Chem.
Rev..

[5] Kim K., Lee S., Nam J.-S., Joo M., Mikladal B., Zhang Q., Kauppinen E. I., Jeon I., An S. (2023). Highly Transparent
and Mechanically Robust Energy-harvestable Piezocomposite with Embedded
1D P­(VDF-TrFE) Nanofibers and Single-walled Carbon Nanotubes. Adv. Funct. Mater..

[6] Chorsi M. T., Le T. T., Lin F., Vinikoor T., Das R., Stevens J. F., Mundrane C., Park J., Tran K. T. M., Liu Y. (2023). Highly
piezoelectric, biodegradable, and flexible
amino acid nanofibers for medical applications. Sci. Adv..

[7] Li T., Yuan Y., Gu L., Li J., Shao Y., Yan S., Zhao Y., Carlos C., Dong Y., Qian H. (2024). Ultrastable piezoelectric
biomaterial nanofibers and fabrics as an
implantable and conformal electromechanical sensor patch. Sci. Adv..

[8] Wu W., Wang L., Li Y., Zhang F., Lin L., Niu S., Chenet D., Zhang X., Hao Y., Heinz T. F. (2014). Piezoelectricity
of single-atomic-layer MoS2 for energy conversion
and piezotronics. Nature.

[9] Wang H. S., Hong S. K., Han J. H., Jung Y. H., Jeong H. K., Im T. H., Jeong C. K., Lee B.-Y., Kim G., Yoo C. D., Lee K. J. (2021). Biomimetic
and flexible piezoelectric
mobile acoustic sensors with multiresonant ultrathin structures for
machine learning biometrics. Sci. Adv..

[10] Shi J., Zhao P., Wang X. (2013). Piezoelectric-Polarization-Enhanced
Photovoltaic Performance in Depleted-Heterojunction Quantum-Dot Solar
Cells. Adv. Mater..

[11] Wang S., Li Y., Yu M., Li Q., Li H., Wang Y., Zhang J., Zhu K., Liu W. (2024). High-performance cryo-temperature
ionic thermoelectric liquid cell developed through a eutectic solvent
strategy. Nat. Commun..

[12] Dinulovic D., Brooks M., Haug M., Petrovic T. (2015). Rotational
Electromagnetic
Energy Harvesting System. Phys. Procedia.

[13] Chamanian S., Uluşan H., Zorlu Ö., Baghaee S., Uysal-Biyikoglu E., Külah H. (2016). Wearable battery-less wireless sensor network with
electromagnetic energy harvesting system. Sens.
Actuators A: Phys..

[14] Chun J., Ye B. U., Lee J. W., Choi D., Kang C.-Y., Kim S.-W., Wang Z. L., Baik J. M. (2016). Boosted
output performance
of triboelectric nanogenerator via electric double layer effect. Nat. Commun..

[15] Liu J., Goswami A., Jiang K., Khan F., Kim S., McGee R., Li Z., Hu Z., Lee J., Thundat T. (2018). Direct-current triboelectricity generation
by a sliding
Schottky nanocontact on MoS2 multilayers. Nat.
Nanotechnol..

[16] Ryu H., Lee J. H., Khan U., Kwak S. S., Hinchet R., Kim S.-W. (2018). Sustainable
direct current powering a triboelectric
nanogenerator via a novel asymmetrical design. Energy Environ. Sci..

[17] Cheng L., Xu Q., Zheng Y., Jia X., Qin Y. (2018). A self-improving triboelectric
nanogenerator with improved charge density and increased charge accumulation
speed. Nat. Commun..

[18] Gao Y., Liu D., Li Y., Liu J., Zhou L., Li X., Zhao Z., Li S., Yang P., Wang Z. L., Wang J. (2023). Achieving high-efficiency triboelectric nanogenerators by suppressing
the electrostatic breakdown effect. Energy Environ.
Sci..

[19] Choi Y. S., Kim S. K., Smith M., Williams F., Vickers M. E., Elliott J. A., Kar-Narayan S. (2020). Unprecedented
dipole alignment in
α-phase nylon-11 nanowires for high-performance energy-harvesting
applications. Sci. Adv..

[20] Zhao Z., Zhou L., Li S., Liu D., Li Y., Gao Y., Liu Y., Dai Y., Wang J., Wang Z. L. (2021). Selection
rules of triboelectric materials for direct-current triboelectric
nanogenerator. Nat. Commun..

[21] Zhang L., Liao Y., Wang Y.-C., Zhang S., Yang W., Pan X., Wang Z. L. (2020). Cellulose II Aerogel-Based
Triboelectric Nanogenerator. Adv. Funct. Mater..

[22] Šutka A., Lapčinskis L., Verners O., Ģe̅rmane L., Smits K., Pludons A., Gaidukovs S., Jera̅ne I., Zubkins M., Pudzs K. (2022). Bio-Inspired
Macromolecular Ordering of Elastomers for Enhanced Contact Electrification
and Triboelectric Energy Harvesting. Adv. Mater.
Technol..

[23] Song Y., Mukasa D., Zhang H., Gao W. (2021). Self-Powered Wearable
Biosensors. Acc. Mater. Res..

[24] Wen Z., Yeh M.-H., Guo H., Wang J., Zi Y., Xu W., Deng J., Zhu L., Wang X., Hu C. (2016). Self-powered textile
for wearable electronics by hybridizing fiber-shaped
nanogenerators, solar cells, and supercapacitors. Sci. Adv..

[25] Choi D., Kim D. W., Yoo D., Cha K. J., La M., Kim D. S. (2017). Spontaneous occurrence
of liquid-solid contact electrification
in nature: Toward a robust triboelectric nanogenerator inspired by
the natural lotus leaf. Nano Energy.

[26] Fang Y., Yang X., Lin Y., Shi J., Prominski A., Clayton C., Ostroff E., Tian B. (2022). Dissecting
Biological
and Synthetic Soft–Hard Interfaces for Tissue-Like Systems. Chem. Rev..

[27] Wu H., Wang S., Wang Z., Zi Y. (2021). Achieving ultrahigh
instantaneous power density of 10 MW/m2 by leveraging the opposite-charge-enhanced
transistor-like triboelectric nanogenerator (OCT-TENG). Nat. Commun..

[28] Wang X., Niu S., Yin Y., Yi F., You Z., Wang Z. L. (2015). Triboelectric
Nanogenerator Based on Fully Enclosed Rolling Spherical Structure
for Harvesting Low-Frequency Water Wave Energy. Adv. Energy Mater..

[29] Kim D. Y., Kim H. S., Kong D. S., Choi M., Kim H. B., Lee J.-H., Murillo G., Lee M., Kim S. S., Jung J. H. (2018). Floating buoy-based triboelectric
nanogenerator for
an effective vibrational energy harvesting from irregular and random
water waves in wild sea. Nano Energy.

[30] Xiong J., Cui P., Chen X., Wang J., Parida K., Lin M.-F., Lee P. S. (2018). Skin-touch-actuated
textile-based triboelectric nanogenerator
with black phosphorus for durable biomechanical energy harvesting. Nat. Commun..

[31] Lai Y.-C., Lu H.-W., Wu H.-M., Zhang D., Yang J., Ma J., Shamsi M., Vallem V., Dickey M. D. (2021). Elastic Multifunctional
Liquid–Metal Fibers for Harvesting Mechanical and Electromagnetic
Energy and as Self-Powered Sensors. Adv. Energy
Mater..

[32] Fang Y., Han E., Zhang X.-X., Jiang Y., Lin Y., Shi J., Wu J., Meng L., Gao X., Griffin P. J. (2020). Dynamic
and Programmable Cellular-Scale Granules Enable Tissue-like Materials. Matter.

[33] Hinchet R., Yoon H.-J., Ryu H., Kim M.-K., Choi E.-K., Kim D.-S., Kim S.-W. (2019). Transcutaneous ultrasound energy
harvesting using capacitive triboelectric technology. Science.

[34] Liu X., Wang Y., Wang G., Ma Y., Zheng Z., Fan K., Liu J., Zhou B., Wang G., You Z. (2022). An ultrasound-driven implantable wireless energy harvesting system
using a triboelectric transducer. Matter.

[35] Lee D.-M., Rubab N., Hyun I., Kang W., Kim Y.-J., Kang M., Choi B. O., Kim S.-W. (2022). Ultrasound-mediated
triboelectric nanogenerator for powering on-demand transient electronics. Sci. Adv..

[36] Pang Y., Huang Z., Fang Y., Xu X., Cao C. (2023). Toward self-powered
integrated smart packaging system – Desiccant-based triboelectric
nanogenerators. Nano Energy.

[37] Kim W., Bhatia D., Jeong S., Choi D. (2019). Mechanical energy conversion
systems for triboelectric nanogenerators: Kinematic and vibrational
designs. Nano Energy.

[38] Qiao Y., Chang W., Cheng A. J., Wang J., Zhang H., Sha Z., He S., Zhang J., Peng S., Wang C. H. (2023). Clapping
triboelectric nanogenerators as self-powered, frequency-insensitive
and gravity-independent vibration sensors. Nano
Energy.

[39] Ravichandran A. N., Calmes C., Serres J. R., Ramuz M., Blayac S. (2019). Compact and
high performance wind actuated venturi triboelectric energy harvester. Nano Energy.

[40] Dong B., Zhang Z., Shi Q., Wei J., Ma Y., Xiao Z., Lee C. (2022). Biometrics-protected optical communication
enabled by deep learning–enhanced triboelectric/photonic synergistic
interface. Sci. Adv..

[41] Barman S. R., Chan S.-W., Kao F.-C., Ho H.-Y., Khan I., Pal A., Huang C.-C., Lin Z.-H. (2023). A self-powered multifunctional dressing
for active infection prevention and accelerated wound healing. Sci. Adv..

[42] Zhang X.-S., Han M.-D., Wang R.-X., Zhu F.-Y., Li Z.-H., Wang W., Zhang H.-X. (2013). Frequency-Multiplication High-Output
Triboelectric Nanogenerator for Sustainably Powering Biomedical Microsystems. Nano Lett..

[43] Fang Y., Tian B. (2019). Curving neural nanobioelectronics. Nat. Nanotechnol..

[44] Niu S., Wang Z. L. (2015). Theoretical systems
of triboelectric nanogenerators. Nano Energy.

[45] Niu S., Wang S., Lin L., Liu Y., Zhou Y. S., Hu Y., Wang Z. L. (2013). Theoretical study
of contact-mode triboelectric nanogenerators
as an effective power source. Energy Environ.
Sci..

[46] Zi Y., Niu S., Wang J., Wen Z., Tang W., Wang Z. L. (2015). Standards
and figure-of-merits for quantifying the performance of triboelectric
nanogenerators. Nat. Commun..

[47] Niu S., Liu Y., Zhou Y. S., Wang S., Lin L., Wang Z. L. (2015). Optimization
of Triboelectric Nanogenerator Charging Systems for Efficient Energy
Harvesting and Storage. IEEE Trans. Electron
Devices.

[48] Choi Y. S., Kim S.-W., Kar-Narayan S. (2021). Materials-Related
Strategies for
Highly Efficient Triboelectric Energy Generators. Adv. Energy Mater..

[49] Dharmasena R. D. I. G., Jayawardena K. D. G. I., Mills C. A., Deane J. H. B., Anguita J. V., Dorey R. A., Silva S. R. P. (2017). Triboelectric
nanogenerators: providing a fundamental framework. Energy Environ. Sci..

[50] Niu S., Liu Y., Wang S., Lin L., Zhou Y. S., Hu Y., Wang Z. L. (2014). Theoretical Investigation and Structural Optimization
of Single-Electrode Triboelectric Nanogenerators. Adv. Funct. Mater..

[51] Niu S., Zhou Y. S., Wang S., Liu Y., Lin L., Bando Y., Wang Z. L. (2014). Simulation method for optimizing
the performance of an integrated triboelectric nanogenerator energy
harvesting system. Nano Energy.

[52] Peng J., Kang S. D., Snyder G. J. (2017). Optimization
principles and the figure
of merit for triboelectric generators. Sci.
Adv..

[53] Attia P. M., Grover A., Jin N., Severson K. A., Markov T. M., Liao Y.-H., Chen M. H., Cheong B., Perkins N., Yang Z. (2020). Closed-loop optimization
of fast-charging protocols
for batteries with machine learning. Nature.

[54] Zhu R., Qiu T., Wang J., Sui S., Hao C., Liu T., Li Y., Feng M., Zhang A., Qiu C.-W., Qu S. (2021). Phase-to-pattern
inverse design paradigm for fast realization of functional metasurfaces
via transfer learning. Nat. Commun..

[55] Barnett J. W., Bilchak C. R., Wang Y., Benicewicz B. C., Murdock L. A., Bereau T., Kumar S. K. (2020). Designing exceptional
gas-separation polymer membranes using machine learning. Sci. Adv..

[56] Lin L., Wang S., Xie Y., Jing Q., Niu S., Hu Y., Wang Z. L. (2013). Segmentally
Structured Disk Triboelectric Nanogenerator
for Harvesting Rotational Mechanical Energy. Nano Lett..

[57] Niu S., Liu Y., Wang S., Lin L., Zhou Y. S., Hu Y., Wang Z. L. (2013). Theory of Sliding-Mode
Triboelectric Nanogenerators. Adv. Mater..

[58] Dan Foresee, F. ; Hagan, M. T. Gauss-Newton approximation to Bayesian learning. In Proceedings of International Conference on Neural Networks (ICNN’97) 1997; Vol. 3, pp 1930–1935.

[59] Niu S., Wang S., Liu Y., Zhou Y. S., Lin L., Hu Y., Pradel K. C., Wang Z. L. (2014). A theoretical study of grating structured
triboelectric nanogenerators. Energy Environ.
Sci..

[60] Lundberg, S. M. ; Lee, S.-I. A Unified Approach to Interpreting Model Predictions Adv. Neural Inf. Process Syst., 2017; Vol. 30.

[61] Lundberg S. M., Erion G., Chen H., DeGrave A., Prutkin J. M., Nair B., Katz R., Himmelfarb J., Bansal N., Lee S.-I. (2020). From local explanations
to global
understanding with explainable AI for trees. Nat. Mach. Intell..

[62] Niu S., Liu Y., Chen X., Wang S., Zhou Y. S., Lin L., Xie Y., Wang Z. L. (2015). Theory of freestanding triboelectric-layer-based
nanogenerators. Nano Energy.

