# Supplementary material for: Interpretable Machine Learning for Evaluating Nanogenerators’ Structural Design
Source: ACS Nano. 2025 Apr 7;19(14):14456–66. doi: 10.1021/acsnano.5c02525 (PMC12333421; doi:10.1021/acsnano.5c02525)
Supplement: Supplementary file 1 [file nn5c02525_si_001.pdf]

# Supporting Information

# Interpretable Machine Learning for Evaluating Nanogenerators' Structural Design

*Chi Han[1], Mingyu Jin[2], Fuying Dong[1], Pengchong Xu[3], Xinnian Jiang[1], Sheling T. Cai[1,4], Yuanwen Jiang[5], Yongfeng Zhang[2], Yin Fang[6*] and Simiao Niu[1*]*

[1]Department of Biomedical Engineering, Rutgers University, Piscataway, NJ 08854, USA

[2]Department of Computer Science, Rutgers University, Piscataway, NJ 08854, USA

[3]Department of Electrical and Computer Engineering, Rutgers University, Piscataway, NJ 08854, USA

[4]Department of Electrical and Computer Engineering, University of Illinois Urbana-Champaign, Urbana, IL 61801, USA

[5]Department of Materials Science and Engineering, University of Pennsylvania, Philadelphia, PA 19104, USA

[6] School of Chemical and Biomedical Engineering, Nanyang Technological University, Singapore 637459, Singapore

*Corresponding Authors: Yin Fang: fangyin205209@gmail.com; Simiao Niu: simiao.niu@rutgers.edu

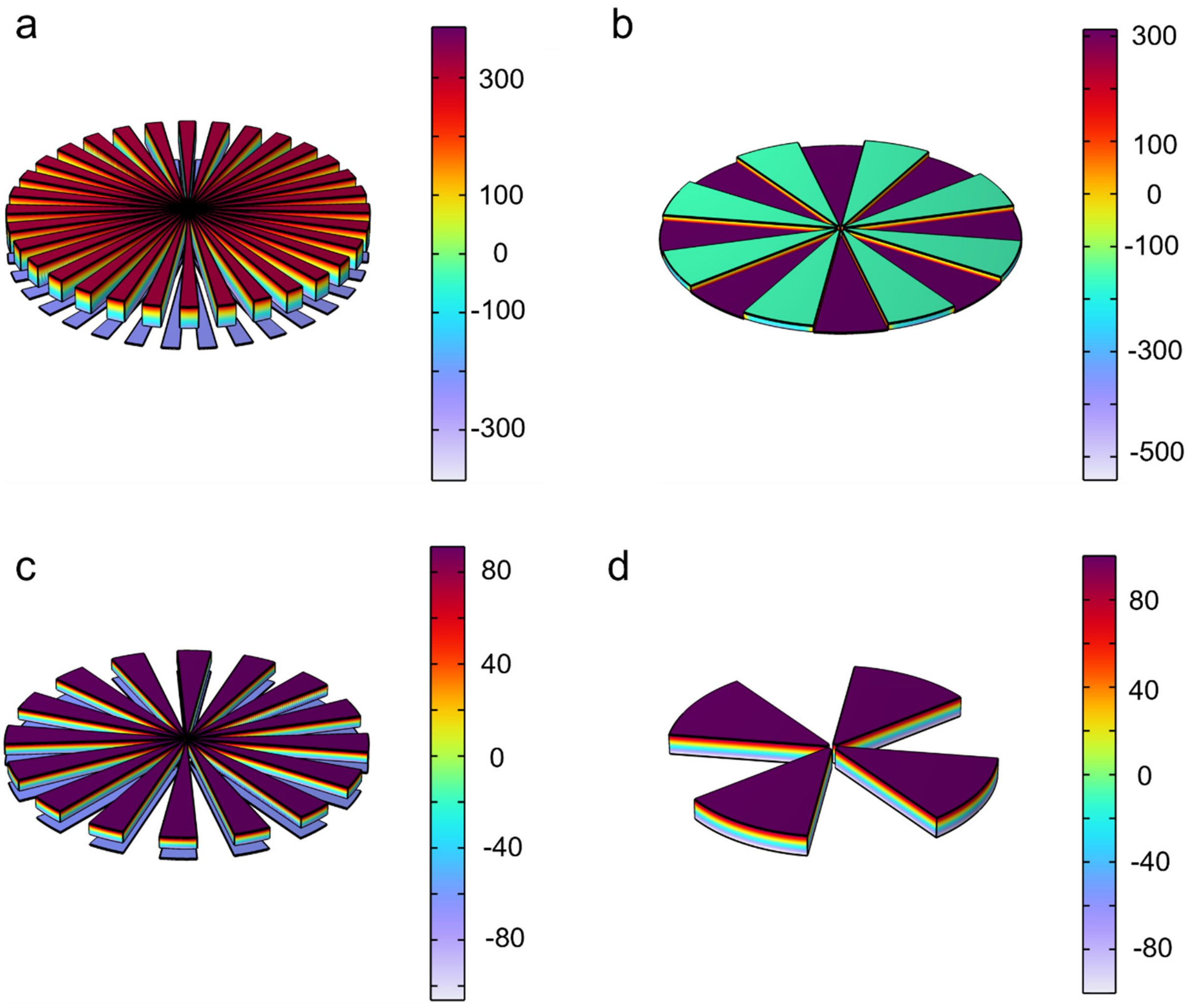


**Figure S1.** COMSOL simulations for disk TENGs, including a. end stage when $n$ = 32, $h$ = 0.001875 m, $d$ = 0.001875 m and $\varepsilon$ = 2, at $Q_{\text{transfer}} = Q_{\text{tribo}}$; b. end stage when $n$ = 8, $h$ = 4.6875E-4 m, $d$ = 0 m and $\varepsilon$ = 1, at $Q_{\text{transfer}}$ = 0; c. start stage when $n$ = 16, $h$ = 9.375E-4 m, $d$ = 9.375E-4 m and $\varepsilon$ = 5, at $Q_{\text{transfer}} = Q_{\text{tribo}}$; d. start stage when $n$ = 4, $h$ = 0.001875 m, $d$ = 0 m and $\varepsilon$ = 10, at $Q_{\text{transfer}} = Q_{\text{tribo}}$.

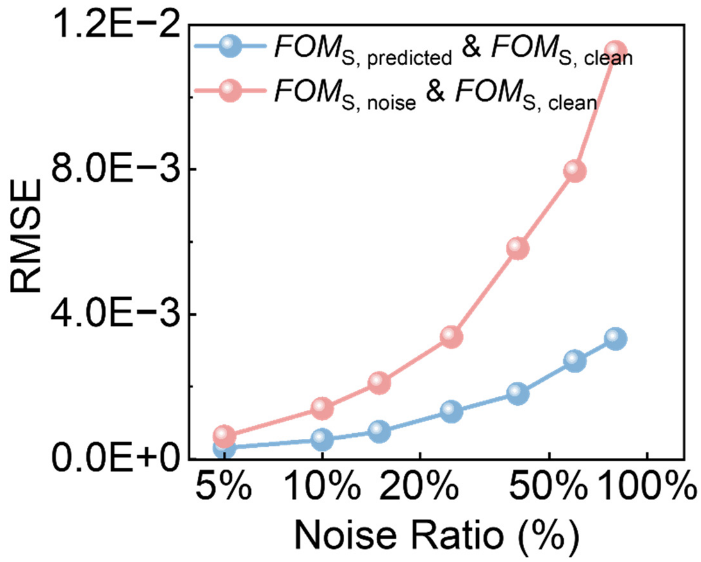


**Figure S2.** RMSE loss values Pearson correlation coefficient *R* between the predicted values and the original values without added noise, and between the original values and the added noise values, obtained by training on the dataset with added noise, with noise ratio ranging from 5% to 80%.

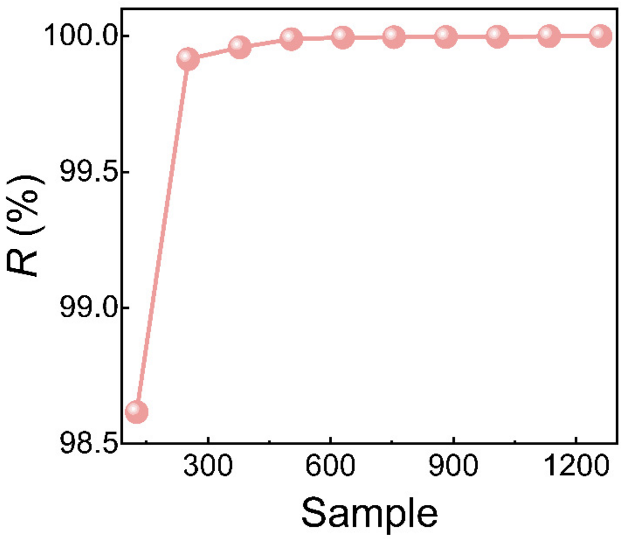


**Figure S3.** Pearson correlation coefficient *R* of ANN-based surrogate model trained on reduced dataset of disk TENG.

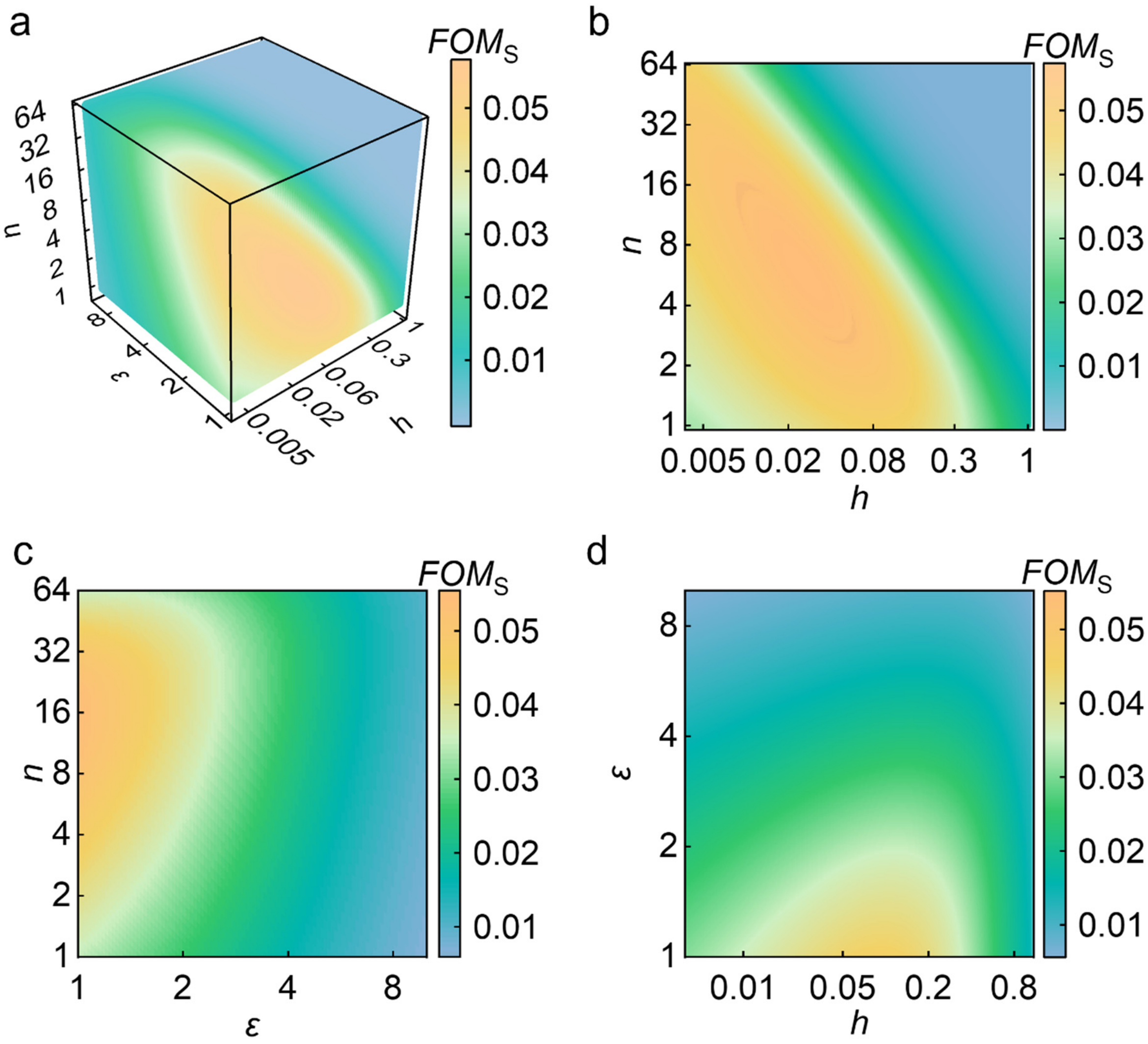


**Figure S4.** a. 3D plot of *FOM*s of dataset generated by ANN-based surrogate model on the *h*-*ε*-*n* coordinate, when $d$ = 0.125; 2D plot of *FOM*s of the dataset generated by ANN-based surrogate model b. on the *h*-*n* coordinate, when $d = 0.125$ and $\varepsilon = 1$, c. on the *ε*-*n* coordinate, when $d = 0.125$ and $h = 0.0078125$, and d. on the *ε*-*n* coordinate, when $d = 0.125$ and $n = 1$.

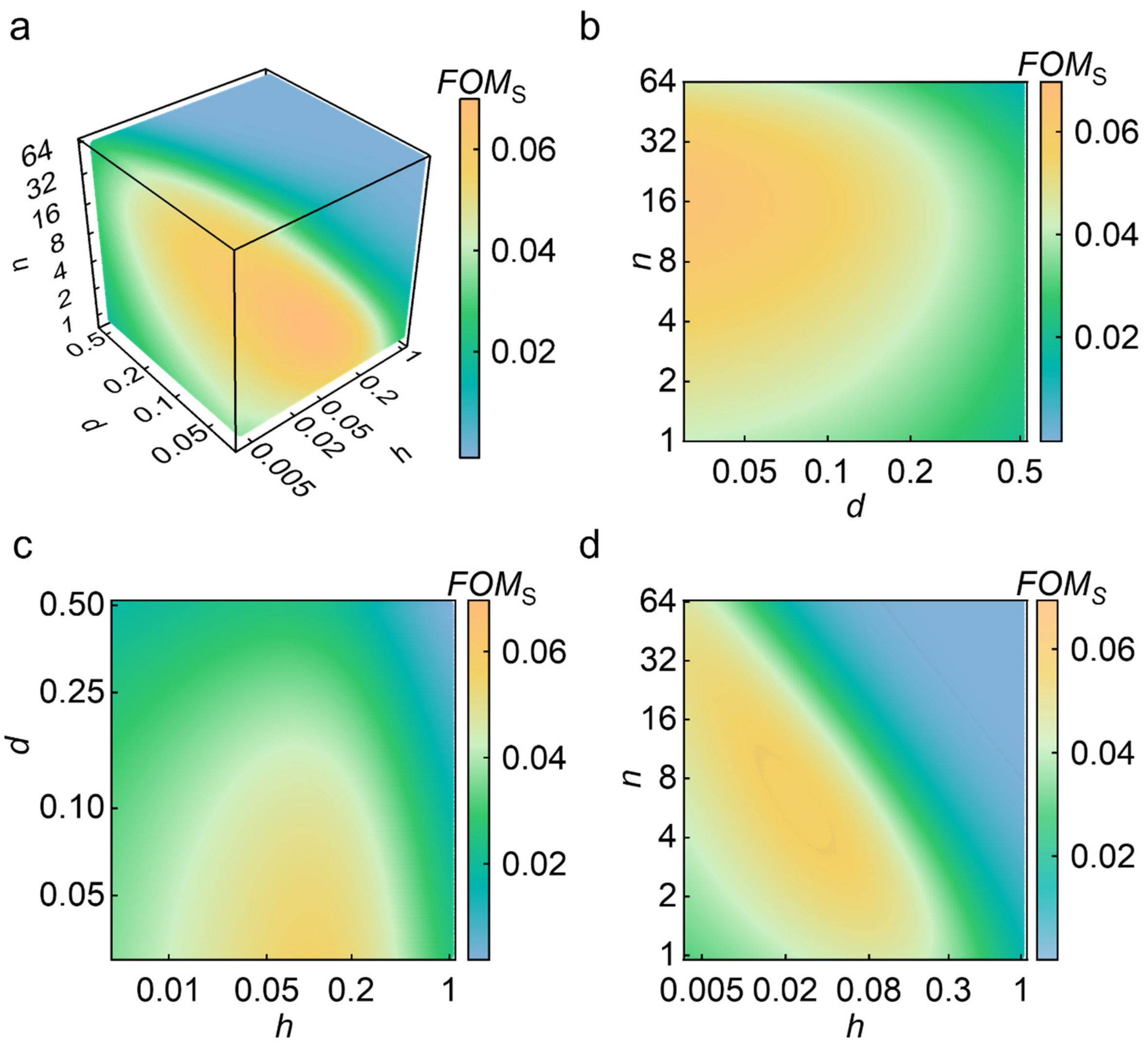


**Figure S5.** a. 3D plot of *FOM*s of dataset generated by ANN-based surrogate model on the *h*-*d*-*n* coordinate, when $\varepsilon = 1$; 2D plot of *FOM*s of the dataset generated by ANN-based surrogate model b. on the *d*-*n* coordinate, when $\varepsilon = 1$ and $h = 0.0078125$, c. on the *h*-*d* coordinate, when $\varepsilon = 1$ and $n = 1$, and d. on the *h*-*n* coordinate, when $\varepsilon = 1$ and $d = 0.125$.

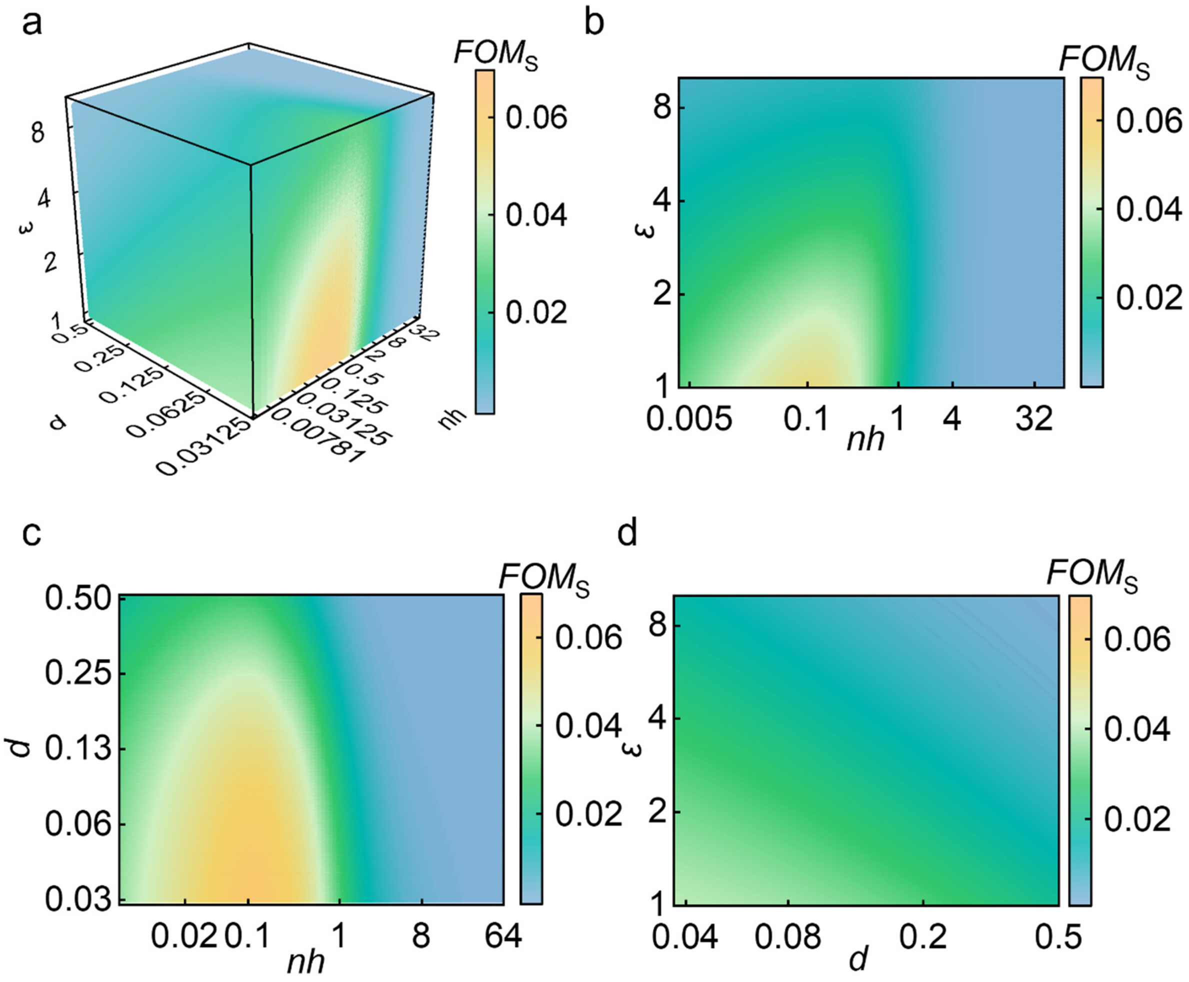


**Figure S6.** a. 3D plot of *FOM*S of dataset generated by ANN-based surrogate model on the *nh*-*d*-*ε* coordinate; 2D plot of *FOM*S of the dataset generated by ANN-based surrogate model b. on the *nh*-*ε* coordinate, when $d$ = 0.125, c. on the *nh*-*d* coordinate, when $\varepsilon$ = 1, and d. on the *d*-*ε* coordinate, when $nh$ = 0.0078125.

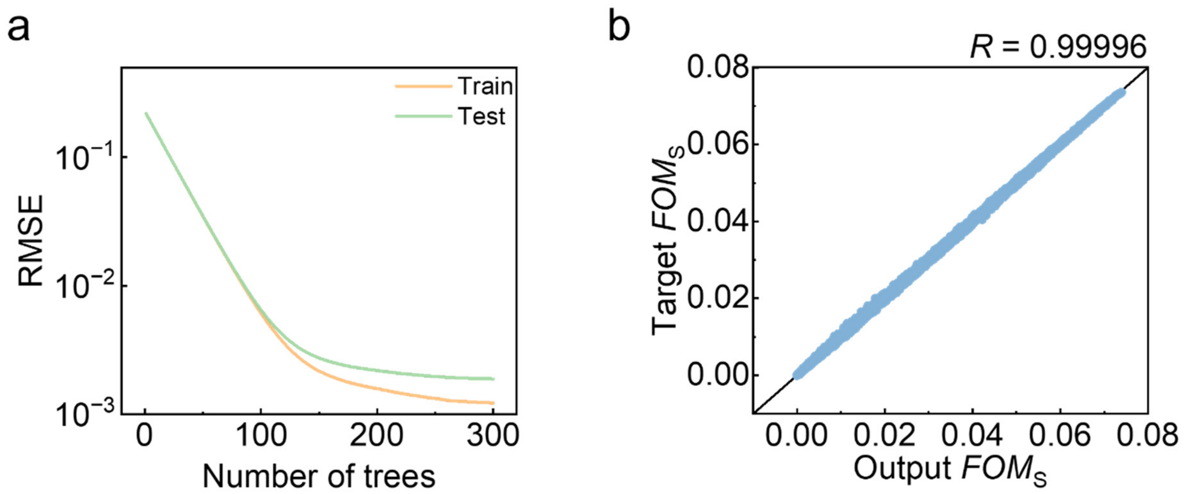


**Figure S7.** a. Relationship between root-mean-square error (RMSE) loss and number of trees plot of train and test dataset for XGBoost model before interaction on disk TENG, and b. Regression plot (with Pearson correlation coefficient $R$) of test dataset for XGBoost model before interaction on disk TENG.

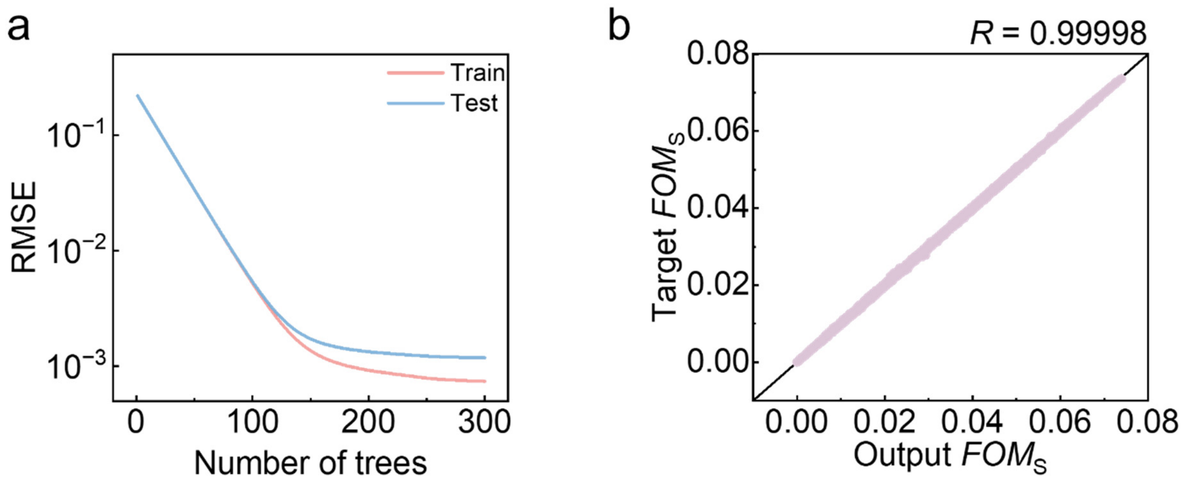


**Figure S8.** a. Relationship between root-mean-square error (RMSE) loss and number of trees plot of train and test dataset for XGBoost model after first interaction on disk TENG, and b. Regression plot (with Pearson correlation coefficient *R*) of test dataset for XGBoost model after first interaction on disk TENG.

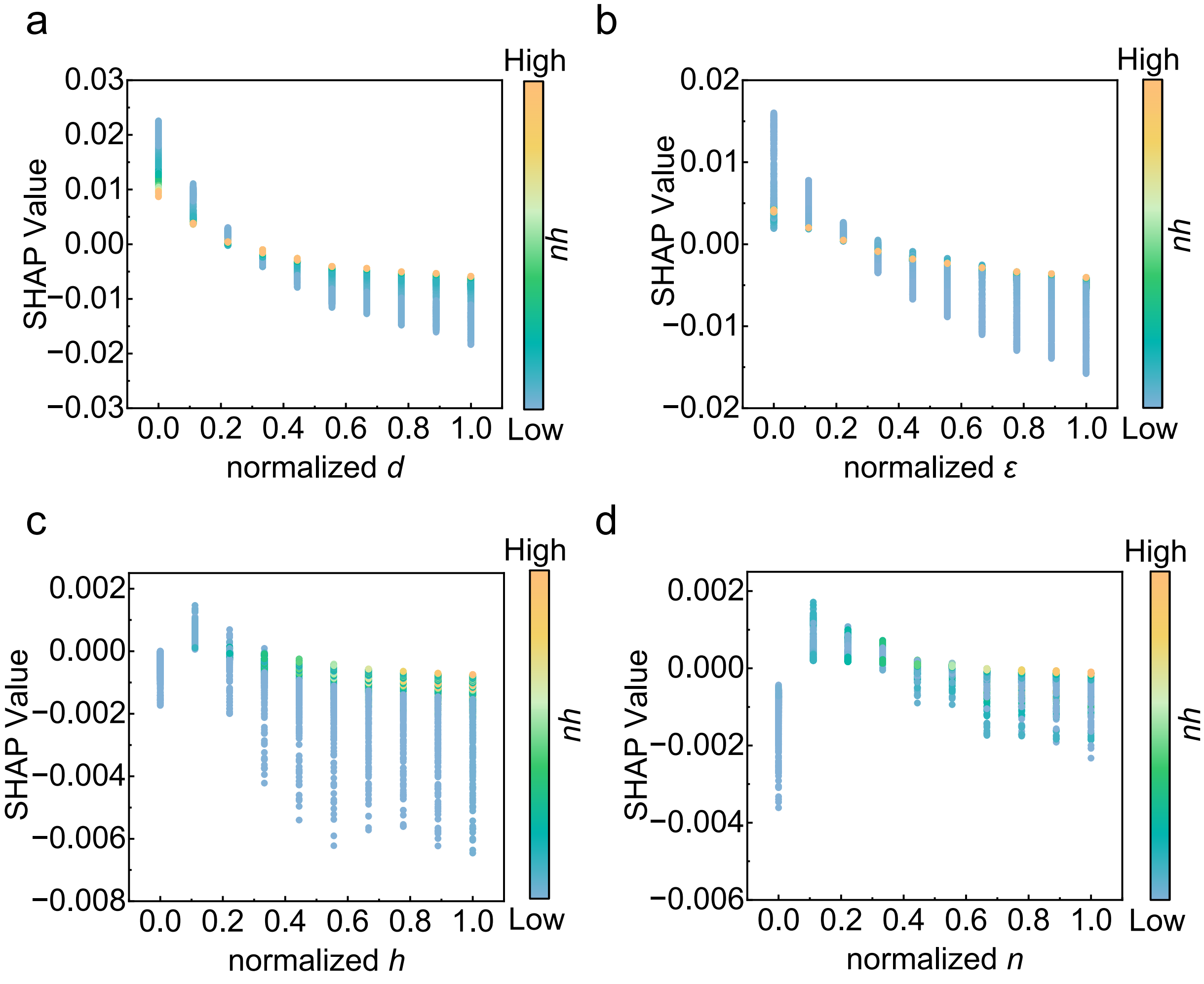


**Figure S9.** SHAP dependence plots of a. normalized feature *d* interacting with normalized feature *nh,* b. normalized feature *ε* interacting with normalized feature *nh,* c. normalized feature *h* interacting with normalized feature *nh,* and d. normalized feature *n* interacting with feature *nh.*

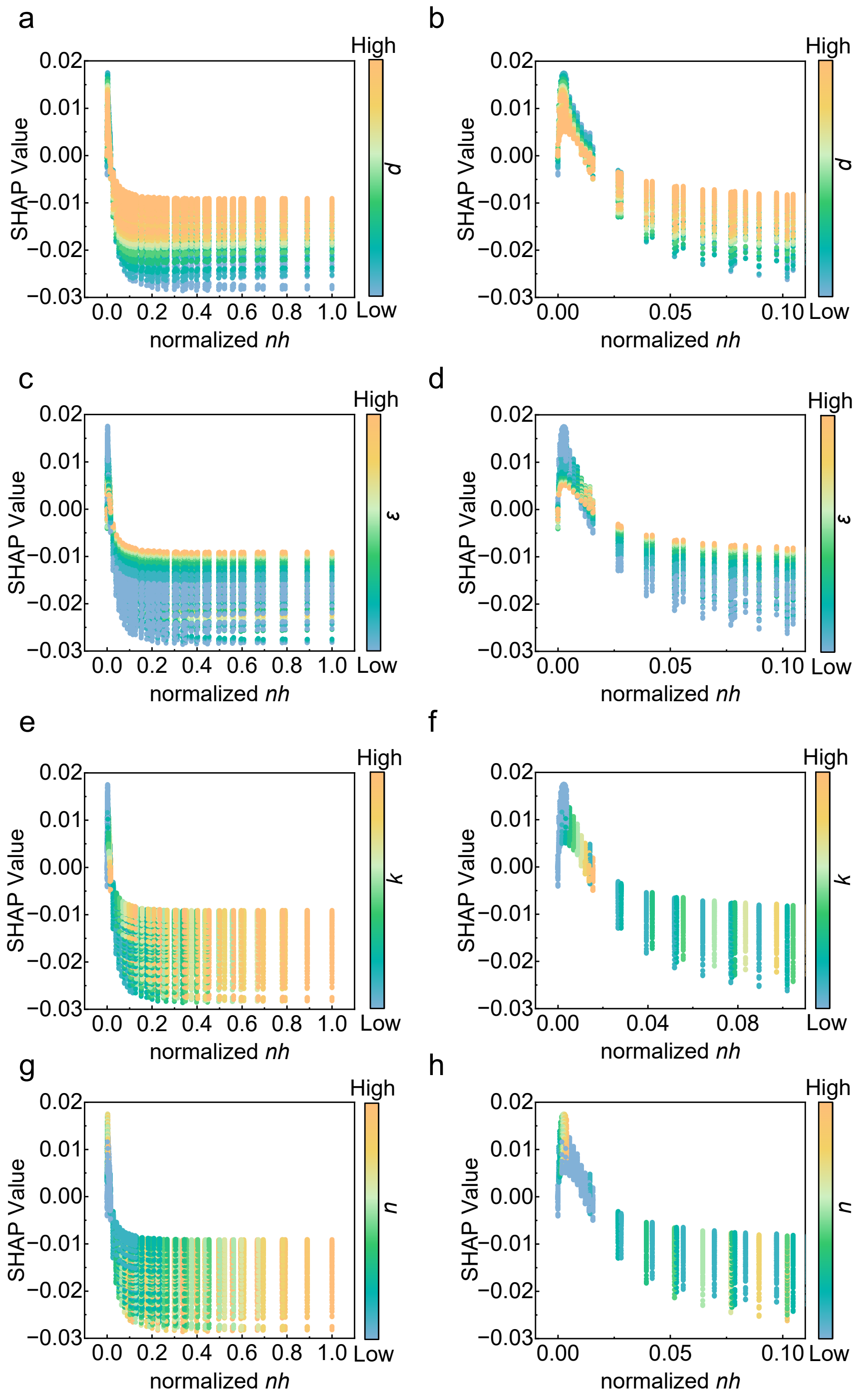


**Figure S10.** SHAP dependence plots of normalized feature *nh* a. interacting with normalized feature *d,* b. in range of 0 to 0.1 interacting with feature *d,* c. interacting with normalized feature *ε,* d. in range of 0 to 0.1 interacting with feature *ε,* e. interacting with normalized feature *h,* f. in

range of 0 to 0.1 interacting with feature *h,* g. interacting with normalized feature *n,* h. in range of 0 to 0.1 interacting with feature *n.*

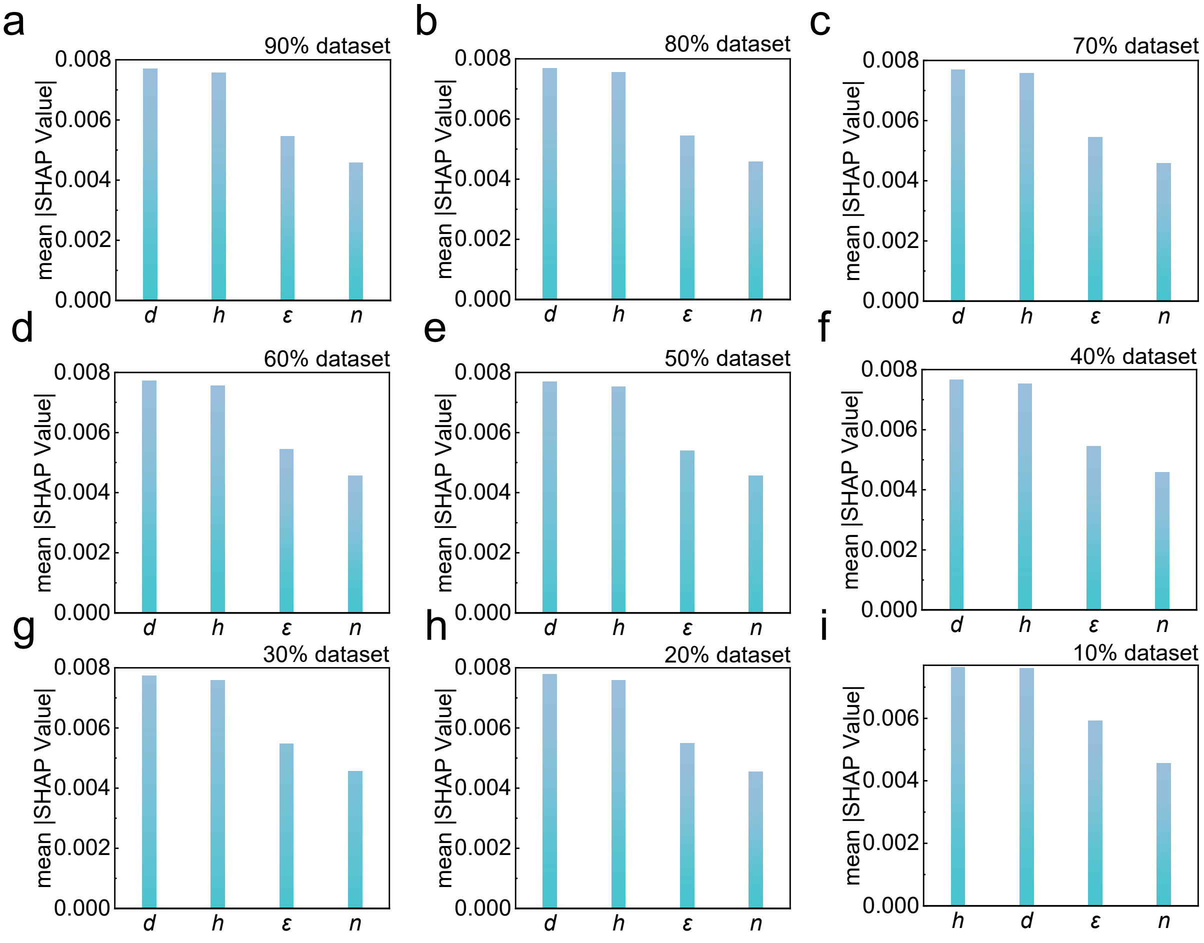


**Figure S11.** Mean absolute SHAP values plot original features on a. original dataset with 10% data reduction, b. original dataset with 20% data reduction, c. original dataset with 30% data reduction, d. original dataset with 40% data reduction, e. original dataset with 50% data reduction, f. original dataset with 60% data reduction, g. original dataset with 70% data reduction, h. original dataset with 80% data reduction, and i. original dataset with 90% data reduction.

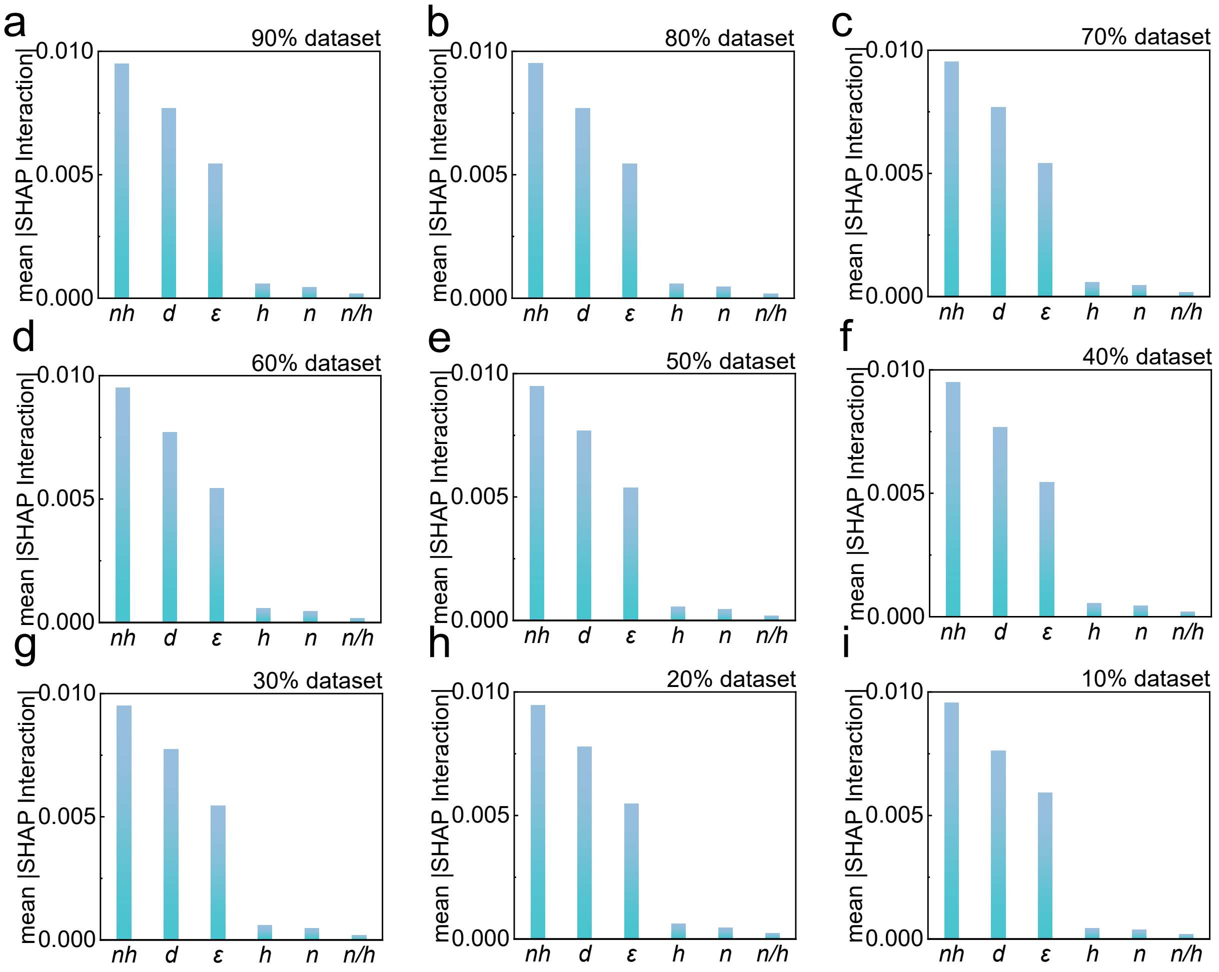


**Figure S12.** Mean absolute SHAP values plot new features on a. dataset after first interaction with 10% data reduction, b. dataset after first interaction with 20% data reduction, c. dataset after first interaction with 30% data reduction, d. dataset after first interaction with 40% data reduction, e. dataset after first interaction with 50% data reduction, f. dataset after first interaction with 60% data reduction, g. dataset after first interaction with 70% data reduction, h. dataset after first interaction with 80% data reduction, and i. dataset after first interaction with 90% data reduction.

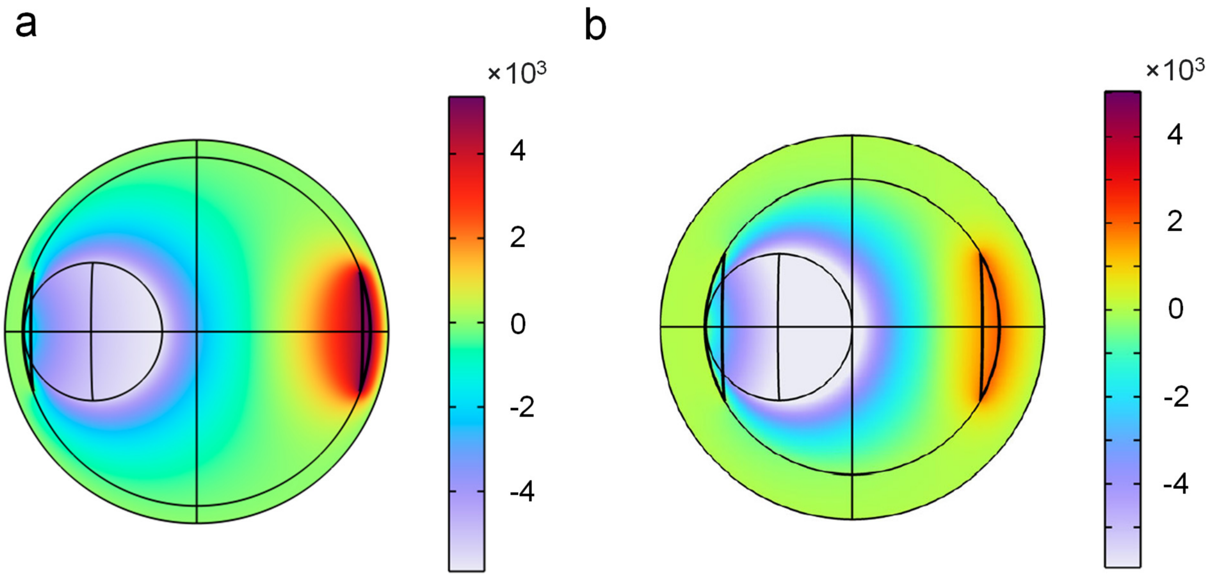


**Figure S13.** COMSOL simulations under symmetric reference state, for spherical TENGs, including a. end (start) stage when $dR = 0.4$, $dH = 0.1$, $\varepsilon_{\text{ball}} = 10$, $\varepsilon_{\text{shell}} = 1$ and $\theta = 10$, at $Q_{\text{transfer}} = 0$; b. end (start) stage when $dR = 0.5$, $dH = 0.3$, $\varepsilon_{\text{ball}} = 5$, $\varepsilon_{\text{shell}} = 5$ and $\theta = 30$, at $Q_{\text{transfer}} = 0$.

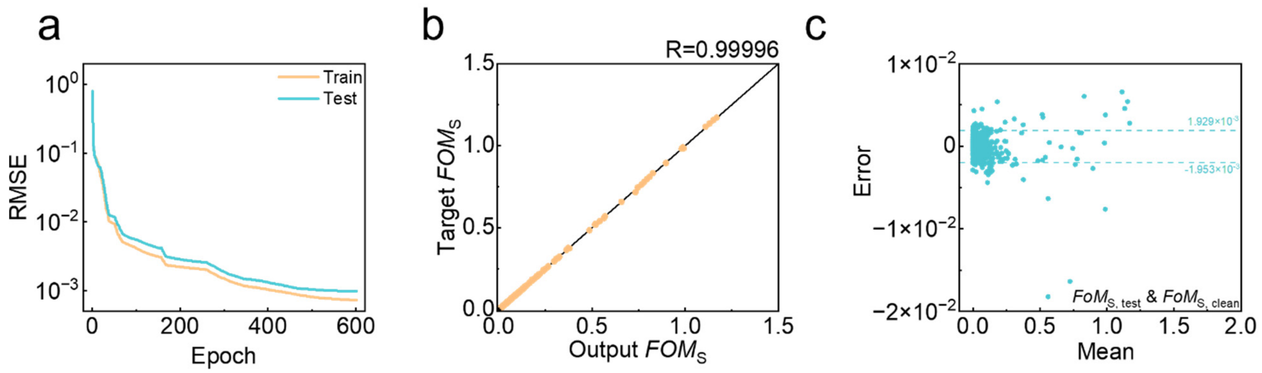


**Figure S14.** a. Relationship between root-mean-square error (RMSE) loss and epoch plot of train and test dataset on spherical TENG, and b. Regression plot (with Pearson correlation coefficient *R*) of test dataset on disk TENG; c. Bland-Altman plot on original dataset without noise.

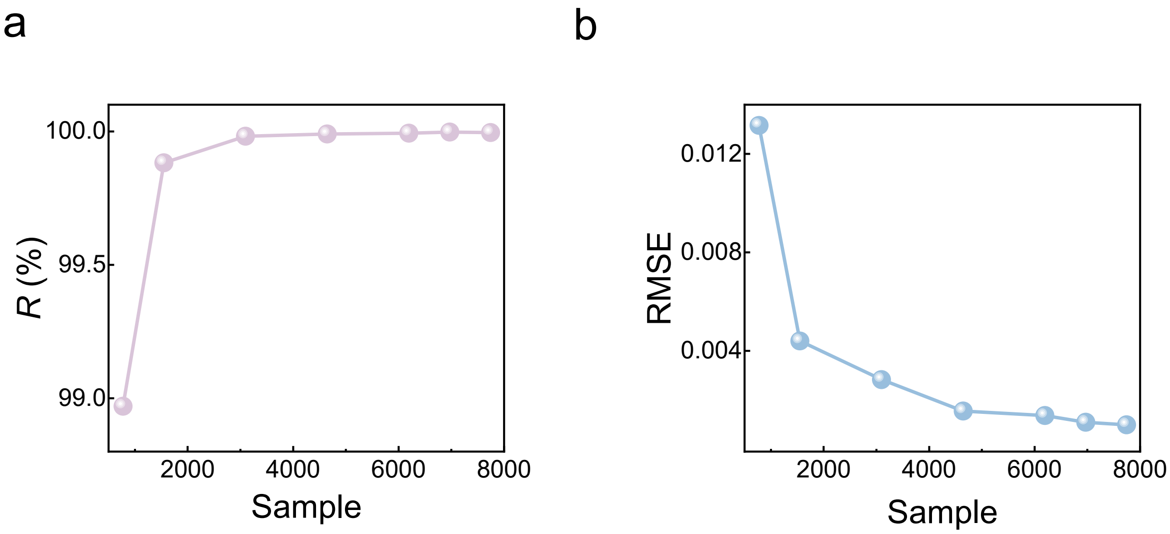


**Figure S15.** a. Pearson correlation coefficient *R* and b. RMSE loss of ANN-based surrogate model trained on reduced dataset of spherical TENG.

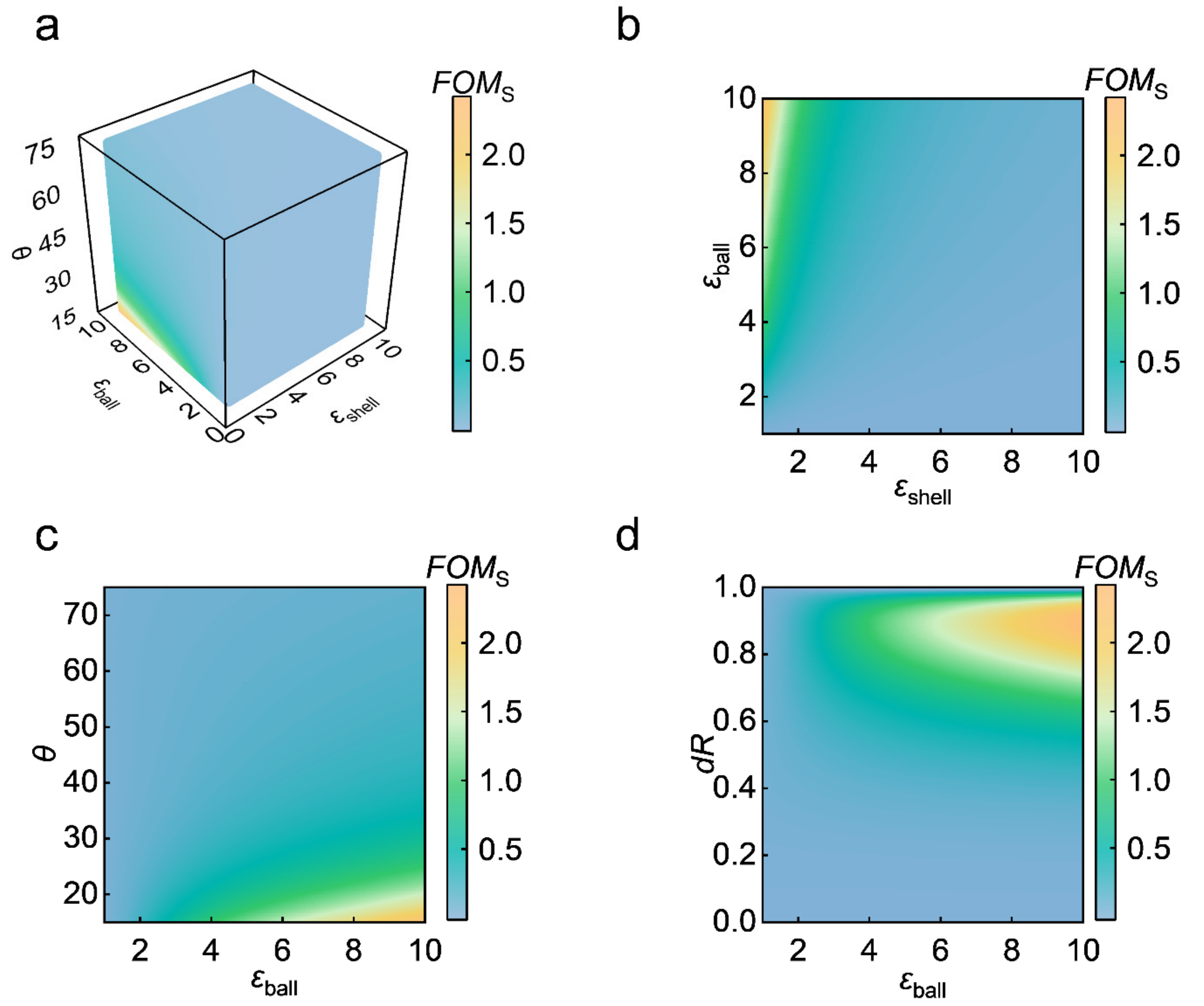


**Figure S16.** 3D plot of *FOM*s of the dataset generated by ANN-based surrogate model on the $\varepsilon_{ball}$-$\varepsilon_{shell}$-$\theta$ coordinate, when $dR = 0.9$ and $dH = 0.2$; a. 2D plot of *FOM*s of the dataset generated by ANN-based surrogate model b. on the $\varepsilon_{shell}$-$\varepsilon_{ball}$ coordinate, when $dR = 0.9$, $dH = 0.2$ and $\varepsilon_{shell} = 1$, c. on the $\varepsilon_{ball}$-$\theta$ coordinate, when $dR = 0.9$, $dH = 0.2$ and $\varepsilon_{shell} = 1$, and d. on the $\varepsilon_{ball}$-$dR$ coordinate, when $\theta = 15$, $dH = 0.2$ and $\varepsilon_{shell} = 1$.

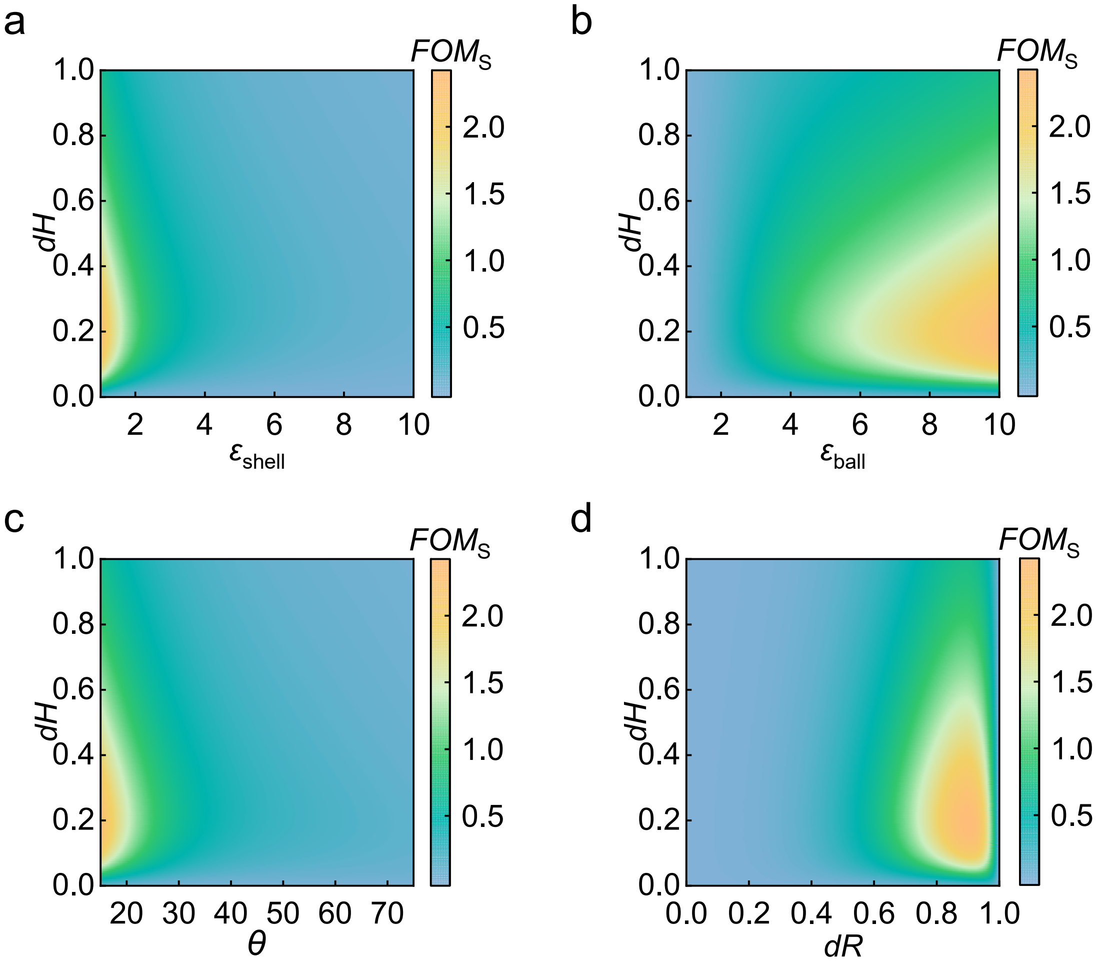


**Figure S17.** 2D plot of *FOM*s of the dataset generated by ANN-based surrogate model a. on the $\varepsilon_{shell}$-$dH$ coordinate, when $dR$ = 0.9, $\theta$ = 15 and $\varepsilon_{ball}$ = 10, b. on the $\varepsilon_{ball}$-$dH$ coordinate, when $dR$ = 0.9, $\theta$ = 15 and $\varepsilon_{shell}$ = 1, c. on the $\theta$-$dH$ coordinate, when $dR$ = 0.9, $\varepsilon_{ball}$ = 10 and $\varepsilon_{shell}$ = 1, and d. on the $dR$-$dH$ coordinate, when $\theta$ = 15, $\varepsilon_{ball}$ = 10 and $\varepsilon_{shell}$ = 1.

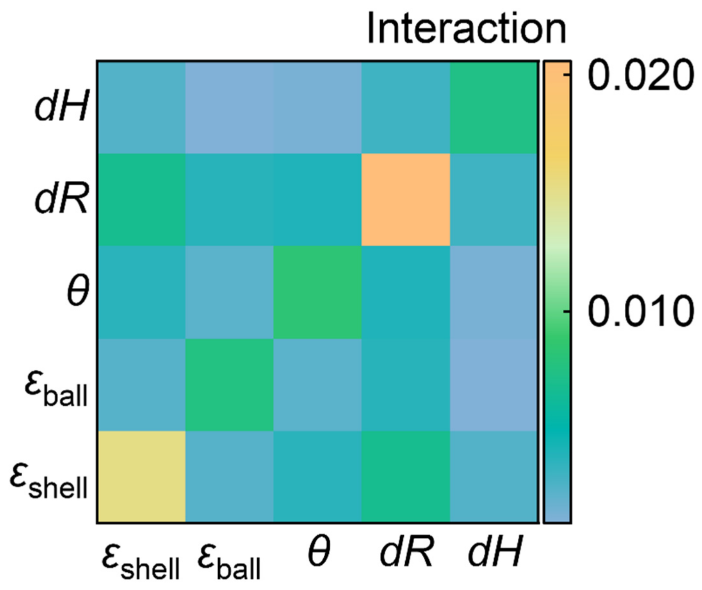


**Figure S18.** Mean absolute SHAP value interaction heatmap of original features on original dataset of spherical TENG.

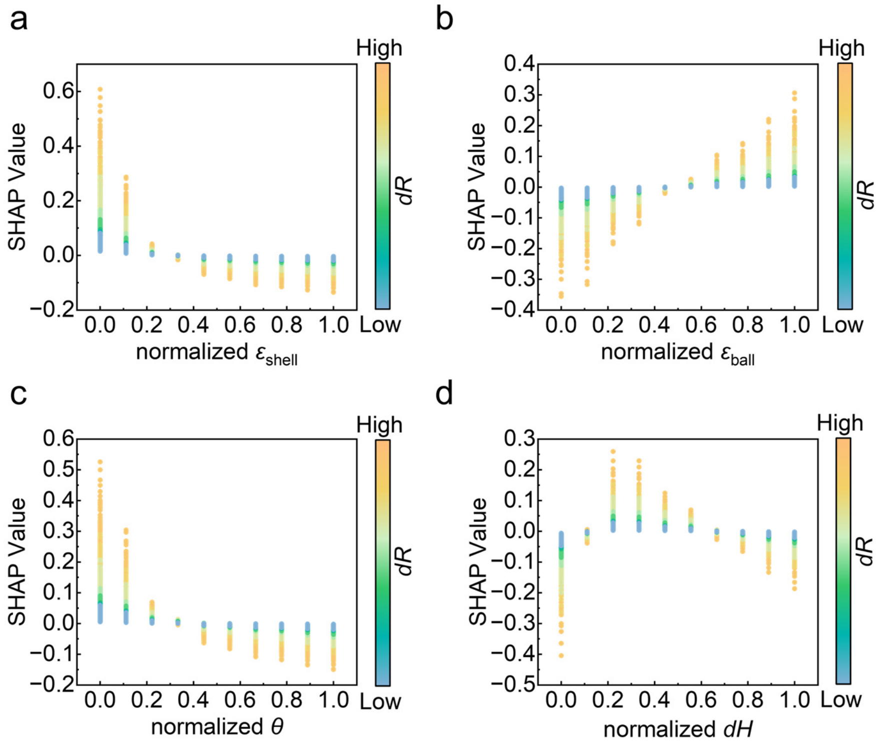


**Figure S19.** SHAP dependence plots of a. normalized feature $\varepsilon_{shell}$ interacting with normalized feature *dR,* b. normalized feature $\varepsilon_{ball}$ interacting with normalized feature *dR,* c. normalized feature $\theta$ interacting with normalized feature *dR,* and d. normalized feature *dH* interacting with feature *dR*.

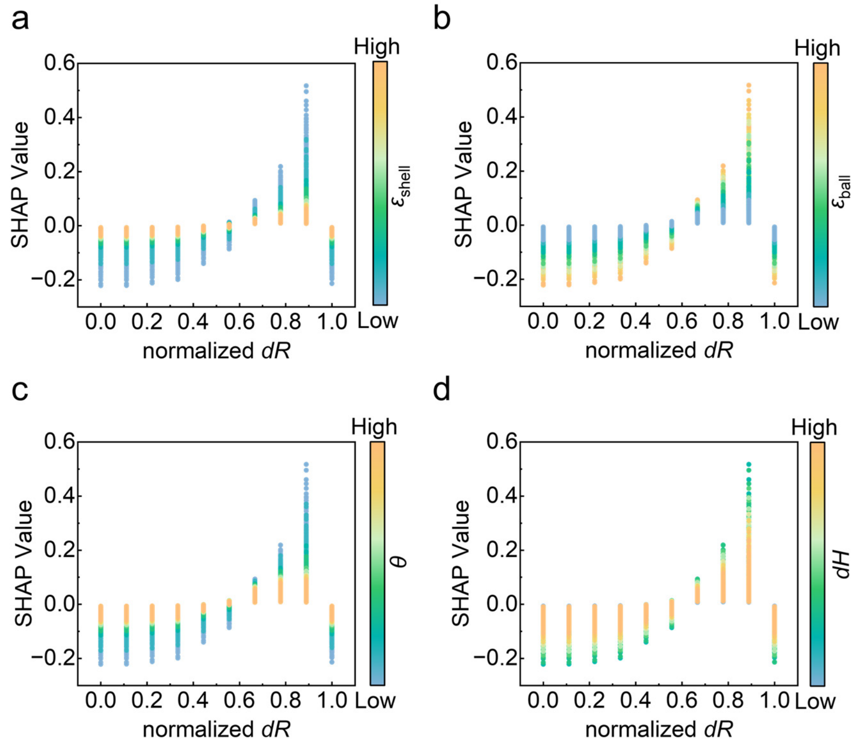


**Figure S20.** SHAP dependence plots of normalized feature *dR* a. interacting with normalized feature $\varepsilon_{shell}$, b. interacting with normalized feature $\varepsilon_{ball}$, c. interacting with normalized feature *θ*, d. interacting with normalized feature *dH*.

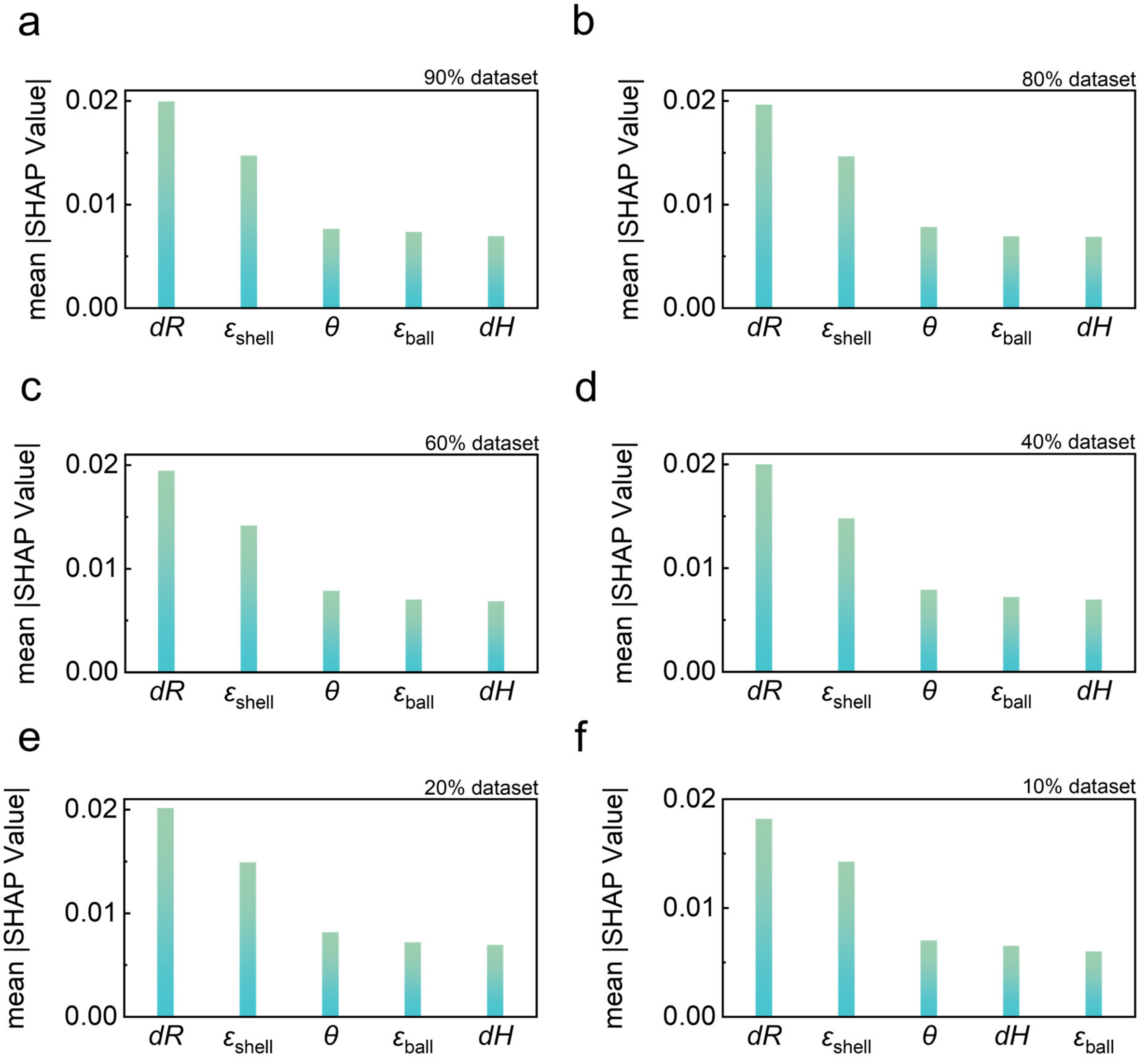


**Figure S21.** Mean absolute SHAP values plot original features on a. original dataset with 10% data reduction, b. original dataset with 20% data reduction, c. original dataset with 40% data reduction, d. original dataset with 60% data reduction, e. original dataset with 80% data reduction, f. and original dataset with 90% data reduction.

| ML algorithms | RMSE Loss | Pearson correlation *R* |
|---|---|---|
| SVM | 5.088E-3 | 0.95391 |
| ANN | 1.089E-4 | 0.99999 |
| Random Forest | 2.893E-3 | 0.98470 |
| XGBoost | 2.248E-3 | 0.99022 |

**Table S1.** Comparison between different ML algorithms for predicting the performance of disk TENGs

| *n* | *ε* | *h* | *d* | *nh* | *n/h* | *FOM*s (4 decimal places) |
|---|---|---|---|---|---|---|
| 8 | 1 | 0.03125 | 0 | 0.25 | 256 | 0.0726 |
| 64 | 10 | 1 | 0.5 | 64 | 64 | 7.3162e-10 |

**Table S2.** Parameters for SHAP local analysis in disk TENGs

| $\varepsilon_{shell}$ | $\varepsilon_{ball}$ | *θ* | *dR* | *dH* | *FOM*s (4 decimal places) |
|---|---|---|---|---|---|

| | | | | | |
|---|---|---|---|---|---|
| 1 | 10 | 15 | 0.9 | 0.2 | 2.4077 |
| 10 | 1 | 75 | 1 | 1 | 0 |

**Table S3.** Parameters for SHAP local analysis in spherical TENGs

**Note S1.**

| Parameters used in the disk TENG simulations | Range |
|---|---|
| Ratio of air gap to dielectric thickness *d* | 0.03125 – 0.5 |
| Ratio of dielectric thickness to disk radius *h* | 0.00390625 – 1 |
| Number of electrode *n* | 1 – 64 |
| Dielectric constant *ε* | 1 – 10 |
| Surface charge density *σ* | 1e-5 [C/m$^2$] |
| Electrode thickness $H_{\mathrm{Al}}$ | 1e-4 [m] |
| Disk Radius *R* | 1.5e-2 [m] |

Table S4. Parameters Specification of disk TENGs

For disk TENG applied here, the *FOM*s can be derived as:

$$FOM_{\mathrm{s}} = \frac{2\varepsilon_0 E_{\mathrm{m}}}{\sigma^2 A x_{\mathrm{max}}} = \frac{\varepsilon_0 Q^2_{\mathrm{scmax,MACRS}}}{\sigma^2 A \frac{\pi R}{n}} \left( \frac{1}{C_{\mathrm{start}}} + \frac{1}{C_{\mathrm{end}}} \right) \quad (S1)$$

$$FOM_{\mathrm{s}} = \frac{2n\varepsilon_0 Q^2_{\mathrm{scmax,MACRS}}}{\sigma^2 \pi^2 R^3} \left( \frac{1}{C_{\mathrm{start}}} + \frac{1}{C_{\mathrm{end}}} \right) \quad (S2)$$

in which the $Q_{scmax,MACRS}$ can be derived from the difference between short circuit transfer charge at end and start stages.

**Note S2.**

| Parameters used in the spherical TENG simulations | Range |
|---|---|
| Relative permittivity of outer shell $\varepsilon_{shell}$ | 1 – 10 |
| Relative permittivity of inner dielectric ball $\varepsilon_{ball}$ | 1 – 10 |
| Central angle of electrode $\theta$ | 30 – 150 |
| Ratio of inner ball's radius to outer shell's inner radius $dR$ | 0 – 1 |
| Ratio of outer shell's thickness to its inner radius $dH$ | 0 – 1 |
| outer shell's inner radius $R$ | 0.05 [m] |
| Surface charge density $\sigma$ | 7e-6 [C/m$^2$] |
| Electrode thickness $H_{Al}$ | 4e-4 [m] |

Table S5. Parameters Specification of spherical TENGs

For spherical TENG applied here, the $FOM_S$ can be derived as:

$$FOM_S = \frac{2\varepsilon_0 E_m}{\sigma^2 V_{shell}} \qquad (S3)$$

in which the $V_{\text{shell}}$ represents the volume of entire spherical TENG. The maximum energy formula $E_m$ can be given as:

$$E_{\text{m}} = \frac{1}{2} Q_{\text{SC,max}} \left( \frac{Q_{\text{SC,max}}}{C(x = x_{\text{max}})} + \frac{Q_{\text{SC,max}}}{C(x = 0)} \right) \tag{S4}$$

As our model is fully symmetric, then we define

$$C(x = x_{\text{max}}) = C(x = 0) = C_0 \tag{S5}$$

Therefore,

$$E_{\text{m}} = \frac{1}{2} Q_{\text{SC,max}}^2 \left( \frac{2}{C_0} \right) = \frac{Q_{\text{SC,max}}^2}{C_0} \tag{S6}$$

The *FOM*S can then be derived as:

$$FOM_{\text{S}} = \frac{2\varepsilon_0 Q_{\text{SC,max}}^2}{\sigma^2 \frac{4}{3}\pi R_{\text{shell}}^3 C_0} \tag{S7}$$

in which the $R_{\text{shell}}$ represents the outer radius of shell. For the model applied here:

$$dR = \frac{R_{\text{ball}}}{R} \tag{S8}$$

$$1 + dH = \frac{R_{\text{shell}}}{R} \tag{S9}$$

$$\eta = \frac{Q_{\text{SC,max}}}{Q_{\text{tribo}}} = \frac{Q_{\text{SC,max}}}{\sigma 4\pi R_{\text{ball}}^2} \tag{S10}$$

in which the $R_{\text{ball}}$ represents the radius of dielectric ball. The *FOM*S can then be simplified as:

$$FOM_s = \frac{24\pi\varepsilon_0 R\eta^2 dR^4}{(1 + dH)^3 C_0} \tag{S11}$$

If $dR = 0$, then $FOM_S$ apparently is 0.

If $dR = 1$, then $FOM_S$ also is 0 as there wouldn't be any transfer charge inside the system, namely $\eta = 0$.

If $dH = 0$, then the capacitance between the electrode and plastic ball shell would be infinity ($C \propto 1/dH$). The ultimate capacitance between the two electrodes $C_0$ should be infinity. Therefore, the $FOM_S$ would be 0.
